# Prediction of regulatory long intergenic non-coding RNAs acting in *trans* through base-pairing interactions

**DOI:** 10.1186/s12864-019-5946-0

**Published:** 2019-07-22

**Authors:** Jules Deforges, Rodrigo S. Reis, Philippe Jacquet, Dominique Jacques Vuarambon, Yves Poirier

**Affiliations:** Department of Plant Molecular Biology, University of Lausanne, Biophore Building, CH-1015 Lausanne, Switzerland

**Keywords:** *Trans*-natural antisense, Long intergenic non-coding RNA, *Arabidopsis thaliana*, Transposon, Gene expression and regulation

## Abstract

**Background:**

Long intergenic non-coding RNAs (lincRNAs) can act as regulators of expression of protein-coding genes. *Trans*-natural antisense transcripts (*trans*-NATs) are a type of lincRNAs that contain sequence complementary to mRNA from other loci. The regulatory potential of *trans*-NATs has been poorly studied in eukaryotes and no example of *trans*-NATs regulating gene expression in plants are reported. The goal of this study was to identify lincRNAs, and particularly *trans*-NATs, in *Arabidopsis thaliana* that have a potential to regulate expression of target genes in *trans* at the transcriptional or translational level.

**Results:**

We identified 1001 lincRNAs using an RNAseq dataset from total polyA^+^ and polysome-associated RNA of seedlings grown under high and low phosphate, or shoots and roots treated with different phytohormones, of which 550 were differentially regulated. Approximately 30% of lincRNAs showed conservation amongst *Brassicaceae* and 25% harbored transposon element (TE) sequences. Gene co-expression network analysis highlighted a group of lincRNAs associated with the response of roots to low phosphate. A total of 129 *trans*-NATs were predicted*,* of which 88 were significantly differentially expressed under at least one pairwise comparison. Five *trans*-NATs showed a positive correlation between their expression and target mRNA steady-state levels, and three showed a negative correlation. Expression of four *trans*-NATs positively correlated with a change in target mRNA polysome association. The regulatory potential of these *trans*-NATs did not implicate miRNA mimics nor siRNAs. We also looked for lincRNAs that could regulate gene expression in *trans* by Watson-Crick DNA:RNA base pairing with target protein-encoding loci. We identified 100 and 81 with a positive or negative correlation, respectively, with steady-state level of their predicted target. The regulatory potential of one such candidate lincRNA harboring a SINE TE sequence was validated in a protoplast assay on three distinct genes containing homologous TE sequence in their promoters. Construction of networks highlighted other putative lincRNAs with multiple predicted target loci for which expression was positively correlated with target gene expression.

**Conclusions:**

This study identified lincRNAs in Arabidopsis with potential in regulating target gene expression in *trans* by both RNA:RNA and RNA:DNA base pairing and highlights lincRNAs harboring TE sequences in such activity.

**Electronic supplementary material:**

The online version of this article (10.1186/s12864-019-5946-0) contains supplementary material, which is available to authorized users.

## Background

The genomes of eukaryotes encode a large number of RNAs that are not coding for proteins. These non-coding RNAs include the well-characterized small RNAs such as microRNAs (miRNAs) and short interfering RNAs (siRNAs). Long non-coding RNAs (lncRNAs) are typically defined as RNA without a defined protein-coding potential transcribed by the RNA polymerase II, thus capped and polyadenylated, and are longer than 200 nucleotides. According to their position relative to neighboring genes lncRNAs can be broadly classified as either (1) overlapping non-coding RNAs (oncRNAs), when the RNA overlaps with the protein-coding gene in the sense direction, (2) intronic non-coding RNAs (incRNAs) when the RNA is completely enclosed in an intron, (3) long intergenic non-coding RNAs (lincRNAs), or (4) *cis*-natural antisense transcripts (*cis*-NATs). *Cis*-NATs are lncRNAs transcribed from the same locus as a sense transcript but generated from the opposite DNA strand. *Cis*-NAT thus display perfect sequence complementarity with at least a portion of the sense transcript, depending on the extent of the overlap. A subset of lincRNAs can be classified as *trans*-NATs when the lncRNAs form only partial sequence complementarity to a sense transcript and is generated from a locus distinct (and sometimes unlinked) from the sense mRNA-coding loci.

Numerous lncRNAs have been found to act as regulators of expression of protein-coding genes in both plants and animals, often acting at the transcriptional level [1–4]. One important mechanism for the modulation of target gene expression by lncRNAs is the modification of the chromatin via DNA methylation or histone modification. For example, repression of transcription of the Flowering Locus C (*FLC*) via recruitment of the Polycomb Repression Complex 2 (PCR2) and changes in histone methylation is influenced by at least three lncRNAs at the *FLC* locus, namely the promoter-derived lncRNA COLDWRAP [5], the incRNA COLDAIR [6] and the *cis*-NAT COOLAIR [7]. LncRNAs can also influence transcription by recruiting elements of the transcriptional machinery, such as in the activation of the pathogen responsive *PR1* gene via the recruitment of a Mediator component by the lincRNA ELF18 [8]. LncRNAs can also influence the steady-state level of target mRNA by post-transcriptional mechanisms. LincRNAs can modify target mRNA splicing by interacting or interfering with the splicing machinery, as described for ASCO in Arabidopsis [9], or influence mRNA stability via interaction with RNA binding proteins, as described for *Staufen* in animals [10]. LncRNAs may act as target mimics for miRNAs, thus preventing cleavage of the miRNA targets. One well described example is the induction of the lncRNA IPS1 by phosphate deficiency in plants, which binds but is not cleaved by miR399, thus preventing down-regulation of the mir399 target PHO2 [11]. LncRNAs can also regulate gene expression by producing siRNA from double-stranded RNA generated by the annealing of lncRNA to a target mRNA [12, 13].

Although the majority of reported effects of lncRNAs on target gene expression implicates changes of steady-state mRNA levels, a few examples of lncRNA influencing target mRNA translation have been described. In animals, lincRNAs have been shown to inhibit translation of target genes by the recruitment of translational repressors or interaction with components of the translation initiation complex [14, 15]. A few *cis*-NATs have also been shown to influence cognate sense mRNA translation, such as the *cis*-NAT to the mouse *UCHL1* gene and the *cis*-NAT to the phosphate exporter gene *PHO1.2* in rice [16, 17]. Recent genome-wide studies in *Arabidopsis thaliana* using either RNAseq of polysome-associated RNA or ribosome footprints has enabled the identification of a number of novel *cis*-NATs associated with changes in cognate target gene translation [18, 19].

While the majority of lncRNAs shown to regulate target gene expression belong to either lincRNAs or *cis*-NATs, very few examples of *trans*-NATs regulating gene expression are reported despite their rather high abundance in eukaryotic genomes. For example, genome-wide analysis of transcripts in Arabidopsis, soybean and rice identified between 1′320 to 25′000 *trans*-NATs [20–23]. Analysis of *trans*-NATs in several animal species indicated that up to 4% of transcriptional units are involved in *trans*-NAT:sense mRNA pairing [24]. Examples of *trans*-NAT influencing target gene expression in animals include the down-regulation of genes involved in nitric oxide (NO) biosynthesis in the snail *Lymnaea stagnalis* by the expression of an antisense transcript of a closely related pseudogene [25], as well as the down-regulation of several genes during mouse oocyte development via siRNA generation from double-stand RNA formation between the antisense transcript of pseudogenes and their protein-coding progenitors [26, 27]. *Trans*-NAT can also be associated with epigenetic modifications, such as demonstrated for the *trans*-NAT to the mammalian pluripotency-associated factor *Oct4*, which recruits a histone methyltransferase to the promoter region of *Oct4*, resulting in suppression of transcription [28]. To our knowledge, no example of *trans*-NATs regulating expression of target gene has been reported in plants.

The main goal of this work was to identify in Arabidopsis lincRNAs, and particularly *trans*-NATs, that have a potential to regulate expression of target genes either at the transcriptional or translational level. We have used a RNAseq dataset from total polyA^+^ RNA and polysome-associated RNA from plants grown under various conditions to find association between lincRNA expression and regulation in *trans* of target gene expression via base-pairing with either a protein-coding mRNA or pairing with DNA of a protein-coding gene. Using a protoplast-based assay, we show the potential for a lincRNA containing a transposon sequence to positively and negatively regulate the expression of multiple genes containing a homologous transposon sequence in their promoters.

## Results

### De novo identification of novel lincRNAs

**Fig. 1:**
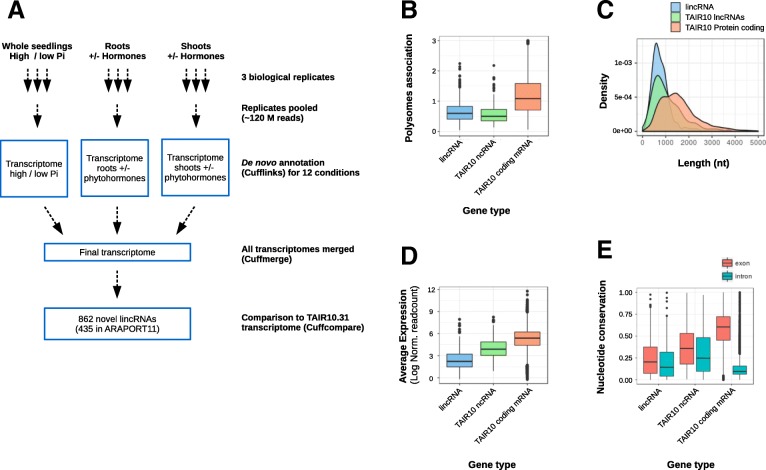
Identification and characterization of novel intergenic transcripts. **a,** Overview of the bioinformatic pipeline used to identify novel lincRNAs. **b,** Boxplot comparing polysome association between novel lincRNAs (blue), TAIR10 lncRNA (green) and TAIR10 protein coding genes (salmon). **c-d,** Plots comparing transcript length (C) and RNA steady-state-level (D) between the 4 categories listed above. **e,** Comparison of the nucleotide conservation across 20 angiosperm genomes (PHASTscore) for exonic (red) and intronic (turquoise) regions between the 3 categories of transcripts listed above

To identify lincRNAs, including *trans*-NATs, that could regulate target gene expression at the transcriptional or translation levels, we analyzed a dataset where steady-state level of polyA^+^ RNAs and polysome-associated mRNAs were measured in *A. thaliana* grown under various conditions (Gene Expression Omnibus accession GSE116553) [19]. Whole *A. thaliana* seedlings were grown in liquid cultures containing a high (1 mM Pi) or a low (100 μM) concentration of inorganic phosphate (Pi), and root or shoots from seedlings grown on agar-solidified medium were treated with various phytohormones, namely auxin (indole acetic acid, IAA), abscisic acid (ABA), methyl-jasmonate (MeJA) or 1-aminocyclopropane-1-carboxylic acid (ACC), a precursor of ethylene. For each experimental condition, steady-state level of polyA^+^ RNA was determined by strand-specific RNAseq and mRNA translation efficiency was analyzed by polysome profiling followed by RNAseq of polysome-associated RNA. Three independent biological replicates for each treatment were analyzed and the dataset includes a total of at least 120 millions of paired-end reads per condition. LincRNAs expressed in the different conditions were identified by the procedure described in the material and methods section and summarized in Fig. 1a. Briefly, transcriptomes were annotated de novo from each of the 12 experimental conditions analyzed, merged and compared to the TAIR10.31 annotation. A total of 1001 lincRNAs were identified, including 862 transcripts that did not overlap any locus annotated in TAIR10.31 (Additional file 9: Table S1). About half the lincRNAs not annotated in TAIR10.31 (435) were later annotated in the Araport11 database [29] and 49% of all identified lincRNAs overlapped a locus already annotated as noncoding transcripts in at least one of the three datasets used for comparison, namely Li et al. [30], Yuan et al. [31], and Bazin et al. [18] (Additional file 1: Figure S1 and Additional file 9: Table S1).

### Conservation amongst plant genomes

Analysis of the 862 lincRNAs not included in TAIR10 showed that approximately one third contained at least one intron and that they had, on average, relatively low polysome association values, similar to annotated TAIR10 non-coding RNAs and significantly lower than TAIR10 protein coding genes (Fig. 1b). They were also smaller, expressed at a lower level and had a weaker genomic sequence conservation (PHASTcons score) compared to annotated protein coding genes (Fig. 1c-e), in agreement with previous reports about non-coding RNAs [31–33]. Studying their conservation amongst plant genomes, we identified a group of 160 and 136 lincRNAs that were conserved beyond the *Arabidopsis* genus and showed high or moderate degree of conservation amongst *Brassicaceae* genomes, respectively (Additional file 2: Figure S2). None of the lincRNAs, however, was clearly conserved outside the *Brassicaceae* group.

### Identification of lincRNAs differentially expressed in response to treatments

The lincRNAs differentially expressed in response to each treatment were identified by pairwise comparison between plants grown on low Pi or treated with hormones and their appropriate controls. In response to low Pi treatment, 58 and 88 lincRNAs were significantly up- and down-regulated, respectively, with a fold change > 2 and adjusted *p* value < 0.1 (Table 1, Additional file 9: Table S1 and Additional file 10: Table S2). With the exception of ABA, fewer lincRNAs were differentially expressed in response to the different hormone treatments. For example, only 4 lincRNAs were up-regulated and 27 down-regulated in IAA treated roots. The strongest difference was observed when untreated root samples were compared to untreated shoots, with 129 lincRNAs more expressed in roots, and 233 less expressed in roots.

**Table 1:** Number of lincRNAs differentially expressed upon different treatments. The experimental conditions compared are indicated in the first column (Treatment) where “ctrl” refers to untreated control. The numbers in brackets indicate the number of lincRNAs present in TAIR10 dataset. The number of lincRNAs up- and down-regulated that are predicted as *trans*-NATs are reported in the columns *trans*-NATs UP and *trans*-NATs DOWN

Treatment	lincRNAs UP (TAIR10)	*trans*-NATs UP	lincRNAs DOWN (TAIR10)	*trans*-NATs DOWN
Low Pi vs High Pi	58 (11)	10	88 (10)	11
Root IAA vs Root ctrl	4 (1)	0	27 (1)	3
Root ABA vs Root ctrl	55 (8)	7	73 (8)	12
Root MeJA vs Root ctrl	4 (1)	0	32 (5)	4
Root ACC vs Root ctrl	0 (0)	0	3 (1)	0
Shoot IAA vs Shoot ctrl	0 (0)	0	1 (0)	1
Shoot ABA vs Shoot ctrl	39 (4)	2	39 (11)	3
Shoot MeJA vs Shoot ctrl	7 (0)	0	4 (3)	0
Shoot ACC vs Shoot ctrl	0 (0)	0	0 (0)	0
Root ctrl vs Shoot ctrl	129 (15)	21	233 (31)	34
Total number of unique genes	192 (32)	31	359 (42)	57

To get insights about the potential function of the differentially expressed lincRNAs analyzed in this study, a weighted gene co-expression network analysis (WGCNA) was constructed from steady-state level values (normalized read count) measured for each gene, coding or non-coding, in each experimental condition analyzed. A total of 17 clusters were obtained, each of them containing protein coding genes as well as lincRNAs sharing similar expression patterns across the 12 experimental conditions (Additional file 3: Figure S3A). For example, the cluster 9 regrouped 1′375 genes up-regulated specifically in response to Pi starvation and expressed more in root than in shoots. In addition to the 1′186 protein coding genes, including 24 associated with the GO term “cellular response to Pi starvation” (GO,0016036), this cluster contained 28 lincRNAs (Additional file 3: Figure S3B). These lincRNAs could thus play a role in the response to Pi starvation. In support of this, a lincRNA with a high expression level belonging in this cluster, XLOC_000075, is a homolog of the AT4, a well characterized lincRNA induced in Pi starvation that impacts Pi homeostasis and acts as a target mimic to the microRNA mir399. This lincRNA has previously been reported by Yuan et al. [31](XLOC_000354) as potentially regulated by PHR1, a transcription factor playing a central role in Pi-deficiency adaptation [34], and by Shin et al. [35] as the AT4 homologue AT4–1.

### Identification of *trans-*NATs correlated with target mRNA expression

To identify *trans-*NATs that could regulate the expression of distant genes via partial *trans*-NAT:mRNA base-pairing, we first looked for complementarity between the set of 1001 lincRNAs identified in this study and protein coding mRNAs. Using the criteria for direct base pair interactions as a complementarity level with an E-value < 1 and an alignment length of at least 100 nucleotides (corresponding approximately to 70% sequence identity for a region of 100 nucleotides), a total of 129 lincRNAs were identified as partially complementary to target mRNAs. Of those *trans*-NATs*,* 88 were significantly differentially expressed with a fold change > 2 and an adjusted *p* value < 0.1 in at least one of the pairwise comparisons performed, with the highest number being differentially expressed by Pi availability, ABA treatment or between roots and shoots (Table 1).

**Fig. 2:**
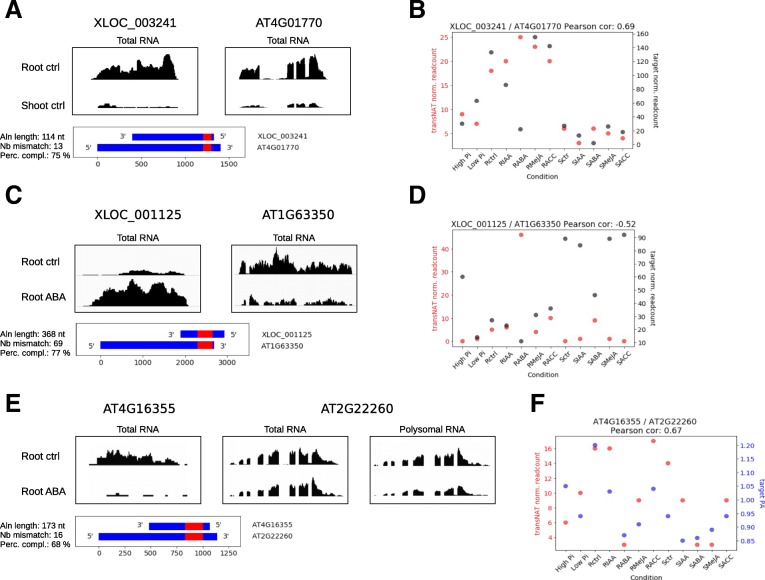
lincRNAs associated with changes of steady-state level or polysome association of potential target genes mRNA. **a** and **b**, Example of a pair showing a positive correlation between lincRNA and target gene mRNA expression. **a,** Density plots showing the density of RNAseq reads in untreated roots (Rctrl) or untreated shoots (Sctrl) for the lincRNA XLOC_003241 (left panel) and its potential target AT4G01770 (right panel). The region of complementarity between the transcripts (blue) is indicated in red on the diagram below. **b,** Correlation plot reporting the steady-state level of XLOC_003241 (red dots) and AT4G01770 (black) transcripts on the Y-axis for each of the 12 experimental conditions analyzed. The Pearson correlation coefficient is indicated on top. **c** and **d,** Example of a pair showing a negative correlation between lincRNA and target gene expression. Same legend as A-B for XLOC_001125 lincRNA and its potential target AT1G63350. **e** and **f,** Example of a pair showing a positive correlation between lincRNA steady-state level and target gene polysome association. **e,** Density plots showing the density of reads from total RNA-seq in untreated roots (Rctrl) or ABA treated roots (RABA) for the lincRNA AT4G16355 (left panel) and its potential target AT2G22260 (center panels). The right panel shows the density of reads from polysomal RNA-seq. The region of complementarity between the transcripts is indicated in red on the diagram below. **f,** Correlation plot reporting the steady-state level of AT4G16355 (red dots) and polysome association of AT2G22260 (blue) transcripts on the Y-axis for each of the 12 experimental conditions analyzed. The Pearson correlation coefficient is indicated on top. For A, C and E, details about the alignment length (Aln length), number of mismatch (Nb mismatch) and percentage of base complementarity (Perc compl) are indicated on the left of each panel showing the region of complementarity between the lincRNAs and the target mRNA

Five *trans*-NATs showed a positive correlation between their expression and target mRNA steady-state levels, and three showed a negative correlation (Table 2, Additional file 11: Table S3). For each pair identified from pair-wise comparison, the Pearson correlation coefficient between *trans-NAT* and target mRNA steady-state level was calculated across the 12 experimental conditions analyzed. As an example of a positive correlation, both XLOC_003241 lincRNA and its potential target AT4G01770 mRNA were up-regulated in untreated roots compared to shoots (FC = 2.79, adj. *P* value = 2.5E-03 and FC = 4.57, adj. *P* value = 1.2E-12 respectively, Table 2, Fig. 2a), with a high Pearson correlation coefficient (0.69) (Fig. 2b). As an example for a negative correlation, XLOC_001125 lincRNA was strongly up-regulated in ABA-treated roots compared to untreated roots (FC = 5.12, adj. *P* value = 1.8E-07) while its predicted target mRNA AT1G63350 was down-regulated (FC = 0.44, adj. *P* value = 1.2E-05, Table 2, Fig. 2c), with a Pearson correlation coefficient of − 0.52) (Fig. 2d). Interestingly, the negative correlation was also observed upon ABA treatment in shoots since XLOC_001125 lincRNA was up-regulated (FC = 2.99, adj. *P* value = 0.01) and AT1G63350 mRNA was significantly down-regulated in the same condition (FC = 0.57, adj. *P* value = 0.043). A predicted RNA-RNA interaction diagram illustrates the extent of sequence complementarity of XLOC_003241-AT4G01770 and XLOC_001125-AT1G63350 (Additional file 4: Figure S4).

**Table 2:** *trans*-NATs correlated with target mRNA steady-state level. For each *trans***-**NAT / target pair, the fold change in RNA steady-state level and associated adjusted *p* value are indicated in columns tNAT_FC and tNAT_pval for transNAT, trgt_FC and trgt_pval for target gene. The experimental conditions compared are indicated in the column “Comparison” where “ctrl” refers to untreated control

*trans*-NAT	Target	tNAT_FC	tNAT_pval	trgt_FC	trgt_pval	Comparison	Correlation
XLOC_003697	AT4G28790	2.8	3.6E-9	2.43	2.9E-8	Root ctrl_Shoot ctrl	Positive
XLOC_000486	AT1G66320	2.27	0.017	3.88	2.2E-4	Root ctrl_Shoot ctrl	Positive
XLOC_002538	AT3G48640	0.06	6.5E-6	0.21	1.3E-6	Root ctrl_Shoot ctrl	Positive
XLOC_001309	AT3G22370	0.33	0.0013	0.28	5.3E-12	Root ctrl_Shoot ctrl	Positive
XLOC_003241	AT4G01770	2.79	0.0025	4.57	1.2E-12	Root ctrl_Shoot ctrl	Positive
XLOC_003681	AT5G41380	0.06	8.6E-17	4.73	3.7E-8	Root ctrl_Shoot ctrl	Negative
XLOC_002538	AT5G66670	0.06	6.5E-6	3.7	3.8E-10	Root ctrl_Shoot ctrl	Negative
XLOC_001125	AT1G63350	5.12	1.8E-7	0.44	1.2E-5	Root ABA_Root ctrl	Negative

### Identification of *trans-NAT*s correlated with target mRNA translation

To identify *trans*-NATs that could potentially influence translation of their target mRNA, we looked for *trans-NAT*:target mRNA pairs where the *trans*-NAT was differentially expressed (fold change > 2 and adjusted *p* value < 0.1) and the target mRNA was differentially associated with polysomes (at least 30% increase of polysome association ratio and adj. *P* value < 0.1). Expression of four *trans*-NATs positively correlated with a change in target mRNA polysome association (Table 3, Additional file 11: Table S3). For example, the TAIR10-annotated lincRNA AT4G16355 was significantly down-regulated in ABA treated roots (FC = 0.3 and adj. *P* value = 0.0013), while its predicted target AT2G22260 was significantly less associated with polysomes (FC = 0.73 and adj. *P* value = 0.067) (Table 3 and Fig. 2e). The Pearson correlation coefficient for this *trans-NAT*- target mRNA pair was 0.67 (Fig. 2f) and a predicted RNA-RNA interaction illustrates the extent of their sequence complementarity (Additional file 4: Figure S4). AT4G16355 has previously been characterized as a lincRNA named *ELENA1* that is induced by the pathogen-associated molecular pattern (PAMP) ELF18 and that regulates the expression of the *Pathogen Response 1* (*PR1*) gene [36, 37].

**Table 3:** *trans*-NATs correlated with target mRNA polysome association. For each *trans***-**NAT / target pair, the fold change in RNA steady-state level and associated adjusted *p* value are indicated in columns tNAT_FC and tNAT_pval for *trans*-NATs, and trgt_FC and trgt_pval for target genes. The fold change in target mRNA polysome association and its associated adjusted *p* value are reported in columns trgt_FC_PA and trgt_pval_PA. The experimental conditions compared are indicated in the column “Comparison” where “ctrl” refers to untreated control

*trans*-NAT	Target	tNAT_FC	tNAT_pval	trgt_FC	trgt_pval	trgt_FC_PA	trgt_pval_PA	Comparison	Correlation
XLOC_002456	AT5G24670	2.06	0.022	0.76	0.031	1.53	0.021	Root ctrl_Shoot ctrl	Positive
XLOC_002963	AT3G27470	2.98	0.0014	1.63	1.0E-6	1.41	0.053	Root ctrl_Shoot ctrl	Positive
XLOC_002528	AT3G10770	4.91	9.6E-9	0.86	0.33	1.3	0.086	Low Pi _High Pi	Positive
AT4G16355	AT2G22260	0.3	0.0013	1	0.98	0.73	0.067	Root ABA_Root ctrl	Positive

### Identification of putative regulatory lincRNAs via complementary to chromatin at target loci

**Fig. 3:**
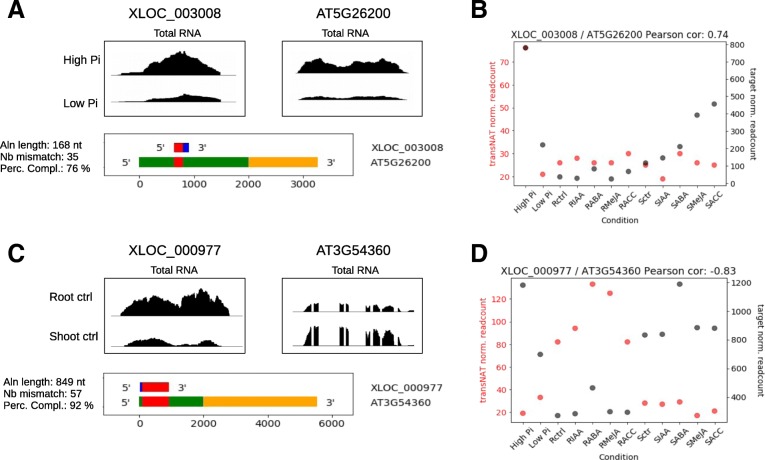
LincRNAs coexpressed or anti-coexpressed with target genes containing a sequence of partial complementarity to the chromatin region including the promoter or gene body. **a** and **b,** Example of a pair showing a positive correlation between lincRNA and target gene expression. **a,** Density plots showing the density of RNAseq reads in seedlings grown in high or low Pi for the lincRNA XLOC_003008 (left panel) and its potential target AT5G26200 (right panel). The region of complementarity between the transcripts is indicated in red on the diagram below, with blue corresponding to RNA of the lincRNA and green and yellow corresponding to the promoter region (2000 nt upstream the transcription start site) and the transcribed region (5′ and 3’UTR, exon and intron) of the target gene, respectively. **b,** Correlation plot reporting the steady-state level of XLOC_ 003008 (red dots) and AT5G26200 (black) transcripts on the Y-axis for each of the 12 experimental conditions analyzed. The Pearson correlation coefficient is indicated on top. **c** and **d,** Example of a pair showing a negative correlation between lincRNA and target gene expression in control roots and shoots. Same legend as A-B for XLOC_000977 lincRNA and its potential target ATG54360. For A and C, details about the alignment length (Aln length), number of mismatch (Nb mismatch) and percentage of base complementarity (Perc compl) are indicated on the left of each panel showing the region of complementarity between the lincRNAs and the target genes

We also looked for lincRNAs that could regulate gene expression in *trans* by Watson-Crick DNA:RNA base pairing with the chromatin at target protein-encoding loci. Such lincRNAs are termed in this study lincRNA-DH for lincRNA-DNA Hybrids. To identify candidate regulatory lincRNA-DH, we looked for homology between lincRNAs and the chromatin region encompassing the complete gene body (5’UTR-exon-intron-3’UTR) plus the promoter region (defined as 2000 bp upstream the annotated transcription start site) for each protein coding gene. A total of 627 lincRNAs showed at least 1 region of homology longer than 100 nucleotides with an E-value < 1 with a protein coding locus, with 214 being differentially expressed in at least one of the 12 pairwise comparisons. Within this later group, changes in steady-state level of 100 were positively correlated with changes in steady-state level of their predicted target, including 64 with Pearson correlation coefficients > 0.6 across the 12 experimental conditions analyzed, while 81 showed negative correlations, including 37 with Pearson correlation coefficients <− 0.6 (Table 4, Additional file 11: Table S3). For example, XLOC_003008 lincRNA and its predicted target AT5G26200 were both strongly down-regulated in seedlings grown in the presence of a low concentration of Pi compared to high Pi samples (FC = 0.36 and 0.34 for XLOC_003008 and AT5G26200 respectively, Pearson correlation = 0.74; Fig. 3a and b). On the opposite, the pair XLOC_000977 / AT3G54360 showed a clear negative correlation between steady-state levels in roots compared to shoots tissues, regardless the hormone treatment (Pearson correlation coefficient = − 0.83, Fig. 3c and d). The global list of lincRNA-DH with their putative chromatin target genes showing a positive or negative correlation included 7 of the 8 pairs predicted for a potential interaction between *trans*-NATs and their target mRNAs described above (Additional file 5: Figure S5).

**Table 4:** lincRNA-DH correlated with target loci steady-state mRNA level. Number of pairs with either a positive or negative correlation between putative lincRNA-DH and predicted target mRNA expression. The experimental conditions compared are indicated in the first column where “ctrl” indicates untreated control. The figures in brackets show the number of those pairs with a Pearson correlation coefficient > 0.6 or < -0.6 across the 12 experimental correlations

Treatment	Positive correlation	Negative correlation
Low Pi vs High Pi	29 (22)	24 (4)
Root ABA vs Root ctrl	13 (5)	5 (1)
Root MeJA vs Root ctrl	5 (2)	1 (0)
Shoot ABA vs Shoot ctrl	8 (1)	6 (0)
Shoot IAA vs Shoot ctrl	1 (1)	0 (0)
Root ctrl vs Shoot ctrl	67 (43)	54 (33)
Total (unique)	100 (64)	81 (37)

**Fig. 4:**
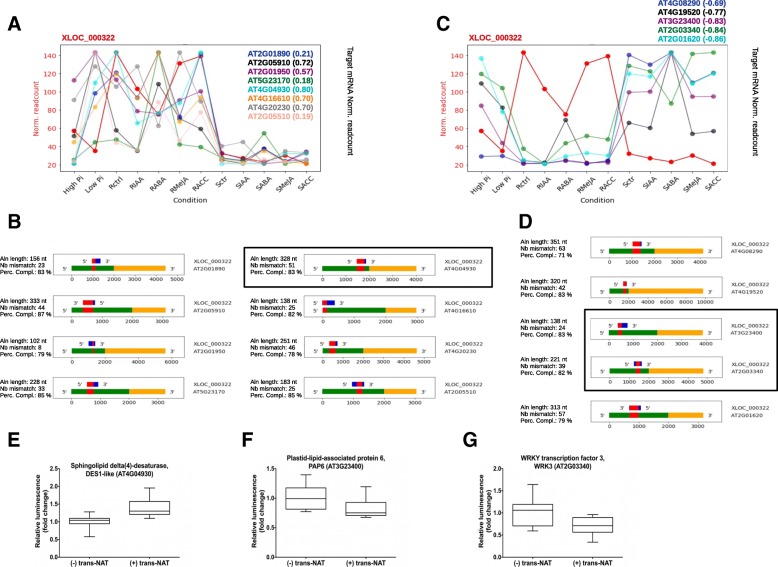
Expression of lincRNA XLOC_000322 influence the expression of several target genes. **a** and **c,** Plot reporting the steady-state level of XLOC_ 000322 (red dots) for each of the 12 experimental conditions analyzed along with the expression of 8 predicted targets genes showing a positive correlation (**a**) and 5 predicted targets showing a negative correlation (**c**). The Pearson correlation coefficient for each gene is indicated in parenthesis beside the gene code. **b** and **d,** Alignment of the XLOC_000322 transcript with the 8 target genes showing positive correlations (**b**) and 5 predicted targets showing a negative correlation (**d**). The region of complementarity between the transcripts is indicated in red on the diagram below, with blue corresponding to RNA of the lincRNA and green and yellow corresponding to the promoter region (2000 nt upstream the transcription start site) and the transcribed region (5′ and 3’UTR, exon and intron) of the target gene, respectively. Details about the alignment length (Aln length), number of mismatch (Nb mismatch) and percentage of base complementarity (Perc compl) are indicated on the left of each panel. **e-g,** Arabidopsis leaf protoplasts were co-transformed with a plasmid combining a predicted target-firefly luciferase (Fluc) fusion and an independent Renilla luciferase (Rluc), along with 0 (− *trans-*NAT) or 2 (+ *trans-*NAT) molar equivalent of an independent plasmid for expression of XLOC_000322. The ratio of Fluc over Rluc activity is plotted for each combination target plasmid in the absence and presence of XLOC_000322. Statistically significant differences based on t-test, *p*-value < 0.05; at least ten biological replicates

Several lincRNA-DH identified as potential regulators had multiple potential target loci predicted (Additional file 11: Table S3). One example that was more closely analyzed was XLOC_000322 lincRNA, which corresponds to a transposon belonging to the Short Interspersed Nuclear Elements (SINE) class of retrotransposon annotated in TAIR10 as AT1TE42205. Expression of XLOC_000322 lincRNA was positively correlated with the expression of 8 predicted targets while it was anti-correlated with expression of 5 predicted target (Fig. 4a-d). A protoplast co-transformation assay was used to validate the effects of XLOC_000322 expression in *trans* on the expression of three targets, namely AT4G04930, AT3G234300 and AT2G03340, which all had high Pearson correlation coefficients. Protoplasts were co-transformed with a plasmid containing the target genes, including 2.0 kbp of their respective promoters, fused to the nano luciferase (nLuc), in the presence or absence of a second plasmid expressing the XLOC_000322 *trans-*NAT. The plasmids containing the target genes fused to nLuc also contained an independent expression cassette for the firefly luciferase (Fluc) that was used as an internal transformation and loading control (see Material and Methods). The ratio nLuc/Fluc was used to assess the effect of XLOC_000322 expression on target gene expression. These protoplasts experiments showed that XLOC_000322 significantly increased the expression of the target gene AT4G04930 (Fig. 4e) while it decreased the expression of AT3G23400 and AT2G03340 (Fig. 4f and g), in agreement with the initial correlations found between expression of XLOC_000322 and steady-state levels of target gene expression.

### lincRNAs coexpressed or anti-coexpressed with neighboring genes

**Fig. 5:**
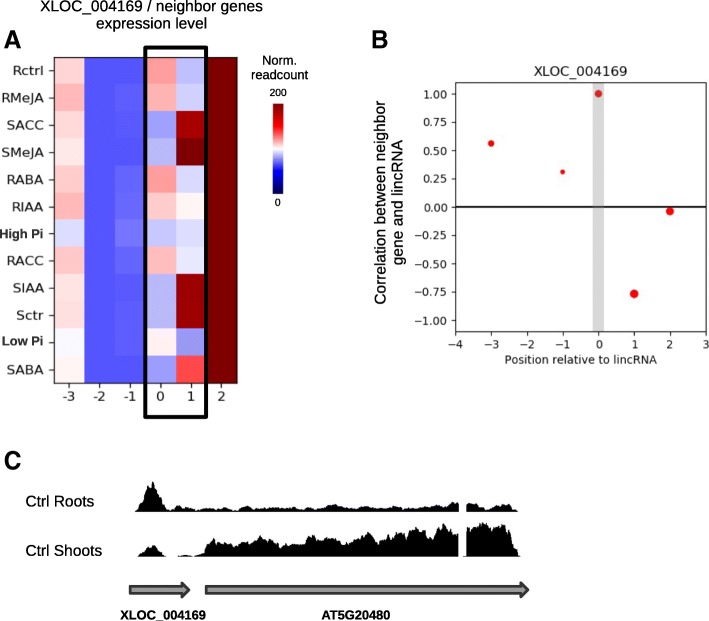
Anti-coexpression between XLOC_004169 lincRNA and its immediate neighboring gene AT5G20480. **a**, Heatmap showing the steady-state level of lincRNA XLOC_004169 (column 0) at its neighbors located within a window of 10,000 nt upstream (genes indexed as − 1 to − 3) or downstream (indexes 1 and 2). The color code indicate the DESeq2 normalized readcount measure for each gene in each of the 12 experimental conditions analyzed. The black frame highlight the lincRNA XLOC_004169 and its immediate downstream neighbor AT5G20480 showing a negative correlation. **b,** Plot reporting the Pearson correlation coefficient calculated from the steady-state levels across the 12 experimental conditions analyzed between the lincRNA and each neighbor gene (indexed by their position relative to lincRNA, similarly to A). **c,** Plot showing the density of reads from total RNA-seq in untreated root (Ctrl Roots) and untreated shoot (Ctrl Shoots) samples. The grey arrows indicate the chromosomic location and orientation of the lincRNA XLOC_004169 and AT5G20480

We also looked for correlation between steady-state levels of lincRNAs and their neighboring genes within a window of 10 kb upstream and downstream each lincRNA. Differential expression of 266 lincRNAs was correlated with changes in steady-state level of at least one neighboring gene in at least one pair-wise comparison (Additional file 11: Table S3). There was a bias towards positive correlation since we identified 224 positive and 142 negative correlation between lincRNA and neighbor gene expression. One example is XLOC_004169 lincRNA which is transcribed from the promoter region of the leucine-rich repeat receptor kinase AT5G20480, immediately upstream its transcription start site and both genes were anti-co-expressed in root compared to shoot tissues (FC = 2.5, ajd. *P* value = 1.3E-04 and FC = 0.21, adj. *P* value = 1.4E-26 for XLOC_004169 and AT5G20480 respectively) (Fig. 5). From the group of lincRNAs positively or negatively correlated with a neighboring gene, 24 were also predicted to interact with the chromatin of these gene, and 2 were predicted to interact with their mRNAs (Additional file 5: Figure S5).

### Network of lincRNAs and target genes

To get a better overview of all the potential interactions between lincRNAs and target gene expression, a network was constructed where lincRNAs and target protein coding genes constituted the nodes, and the different types of potential regulation were represented by edges (Additional file 6: Figure S6A). This representation highlighted several putative *trans-*NATs with multiple predicted targets and complex interactions (Additional file 6: Figure S6B, C). One interesting example is XLOC_000685 lincRNA which has 13 predicted chromatin target loci, the expression of 10 of them being positively correlated and significantly up-regulated in shoots compared to root tissues (Additional file 6: Figure S6C). The genes of four of these target loci belong to the Receptor Like Protein family (RLP23, RLP27, RLP42 and RLP54).

### Links of lincRNAs with miRNA, siRNAs and transposons

LincRNAs were analyzed for the presence of miRNA target sites, miRNA mimic or miRNA precursor sequences (Additional file 9: Table S1). Approximatively 3% were predicted to contain at least one miRNA binding site (31 / 1009), including TAS1A (AT2G27400) and TAS2 (AT2G39681) which were previously shown to be targets for miR173 target [38]. Seven of those lincRNAs predicted to contain at least one miRNA binding site are found in the group of putative regulatory lincRNA-DH via complementary to chromatin at target loci. Seven lincRNAs contained potential miRNA target mimic sequences (Additional file 9: Table S1). One of them, XLOC_000075 (AT4–1), was predicted to contain a miR399 target mimic sequence, as expected for a close homolog of the target mimic AT4 and IPS1 transcripts [11, 35]. In addition, 5 lincRNAs contained sequences homologous to miRNA precursors, 4 of them being later formally annotated at miRNA precursors in Araport11 database. None of the lincRNAs with potential miRNA target mimic sequences or homologous to miRNA precursors have been identified in this study as potentially involved in target gene regulation. (Additional file 9: Table S1).

We also took advantage of 40 publicly available small RNA datasets to analyze the *trans-*NATs capable of forming significant RNA sense-antisense complementarity in relation to siRNAs. Following the procedure described in Yuan et al. [22], we identified 313,448 small reads between 18 and 28 nucleotides long mapping to *trans-NAT*s, most of them being 24 nucleotide long (Additional file 7: Figure S7A-B). The region of *trans-NAT*s with complementary to their putative target showed in average a higher density in small reads than non-complementary sequences (average enrichment score = 4.59, Additional file 7: Figure S7C). Similarly, regions of putative target genes complementary to their predicted *trans*-NAT also showed higher small read densities although the enrichment was weaker (average enrichment score, 1.50) in agreement with previous reports [22, 23]. We identified 49 putative siRNA precursor *trans*-NATs that met the following criteria, at least 5 unique small reads mapped to the region complementary to their predicted target and the read density was at least 2 times higher in complementary than non-complementary region (Additional file 9: Table S1). Only 1 of them was found correlated negatively (XLOC_003681) and 1 positively (XLOC_000486) with the putative target steady-state mRNA level (Table 2).

We also identified 254 lincRNAs (25% of all lincRNAs) with sequences highly homologous to transposable elements (TE) present in the TAIR10 database (Additional file 9: Table S1). Of those, approximately 40% harbored sequences to the RC/Helitron class, with sequences derived from MuDR, Gypsy and Copia being also well represented (Additional file 8: Figure S8). The proportion of TE-lincRNA was enriched to 40% (52 out of 130) in the group of lincRNA-DH with potential binding sites within chromatin of target genes showing a correlation in terms of steady-state level. Similarly, 3 of the 4 putative translation enhancer *trans*-NATs contained TE as well as 3 out of 8 lincRNAs correlated with their predicted target mRNA steady-state level (Additional file 9: Table S1).

## Discussion

This study identified 1001 lincRNAs in Arabidopsis, with more than half differentially regulated either by Pi concentration, phytohormone treatments or between root and shoot. Identification of the functional role and mode of action of lincRNAs is an important challenge considering their high number in eukaryotic genomes. One approach relies on identifying gene networks that are co-regulated with lincRNAs, such as revealed by WGCNA. Such an analysis identified a cluster of genes and lincRNAs that are co-regulated in roots by Pi deficiency (Additional file 3: Figure S3). This cluster included genes encoding proteins well known to be important players in Pi homeostasis, such as the phosphate importer *PHT1;2* and the Pi exporter *PHO1* [39], genes involved in galactolipid synthesis and lipid remodeling under Pi deficiency (*MGD2*, *DGD2*, *PAH1* and *NPC3*) [40], several members of the purple acid phosphatases family (*PAP12*, *PAP22*, *PAP14*) [41] and as well as the *NIGT1/HRS1* gene encoding a transcription factor involved in phosphorus and nitrogen nutritional regulation [42]. This same cluster included the lincRNA IPS1 and two close homologues (AT4 and XLOC000075), which are target mimics to mir399, playing a central role in Pi sensing and adaptation [11]. Further analysis of other lincRNAs associated with this cluster is thus likely to reveal other important lincRNA acting in the adaptation of plants to Pi deficiency.

While WGCNA and similar analysis may reveal in which pathways or biological processes lincRNAs may contribute, it does not necessarily identify the target genes that are directly regulated by lincRNAs. Numerous lincRNAs have been shown to control the expression of closely associated genes via the local recruitment of chromatin modifying protein, such as the PCR2 complex [1–4]. In this context, analysis of the expression pattern of protein-coding genes that are closely linked to lincRNAs may be very fruitful. This study identified 224 positive and 142 negative correlations between lincRNAs and neighboring genes expression (Additional file 11: Table S3). The bias towards positive correlations may, to some extent, reflect changes in chromatin state of the whole region, affecting access of the transcription machinery to both lincRNA and neighboring genes instead of a direct effect of lincRNA expression on the associated genes. The negative correlations, on the other hand, might indicate a direct negative regulation of lincRNAs on neighboring genes. The negative correlation we observed between expression of the lincRNA XLOC_004169 and the neighboring gene AT5G20480 may be associated with transcriptional interference, with transcription of the lincRNA within the promoter region of AT5G20480 inhibiting recruitment of transcription activator(s) required for optimal expression of the gene. A well described example of transcriptional interference in *Saccharomyces cerevisae* is the expression of the *SRG1* lincRNA from the promoter region of the *SER3* gene, resulting in transcriptional suppression of the protein-coding gene [43].

An interesting aspect of the mode of action of lncRNA on target gene expression relates to how specificity is generated. For *cis*-NATs, base-pairing between the sense and antisense RNA is likely to be important even when the mechanism of regulation does not involve the generation of siRNAs. The fact that the specific impact of the *cis*-NATs to the rice *PHO1.2* or mouse *UCHL1* gene on cognate sense mRNA translation can occur when the lncRNAs are expressed in *trans* support a role for direct lincRNA:target mRNA base paring [16, 17]. The same is likely to be true also for the interaction of several *trans*-NATs to their target genes. Our study identified a total of 88 *trans*-NATs that were differentially regulated. Of those, the expression 5 and 3 *trans*-NATs was found to be negatively and positively associated, respectively, with the steady-state mRNA level of their potential target genes. Furthermore, the expression of 4 *trans*-NATs was found positively associated with an increase in target gene mRNA polysome association, indicative of increased mRNA translation. None of the *trans*-NATs associated with changes in target gene steady-state mRNA or polysomal mRNA levels harbored potential miRNA target mimic sequences and only two were associated with the generation of siRNA, one for a positive association and one for a negative association with steady-state mRNA level. Although the cause-and-effect relationship between *trans*-NAT expression and changes in target gene transcription or translation still needs to be experimentally validated, these data indicate that the miRNA or siRNA pathways are unlikely to contribute to the regulation of target gene expression by these *trans*-NATs.

Most target genes potentially regulated by *trans*-NATs found in this study have no or poorly defined function. However, the potential translation regulatory *trans*-NAT At4g16355 (Fig. 2e) is a lincRNA previously named *ELENA1* which is induced by the PAMP ELF18 and interacts with the Mediator subunit 19a to increase expression of genes involved in plant immunity, such as PR1 [36, 37]. The potential target of *ELENA1*, AT2g22260, is coding for a protein involved in DNA demethylation [44]. Interestingly, extensive changes in DNA methylation patterns are associated with the response of Arabidopsis to bacterial and fungal plant pathogens [45, 46]. The fact that *ELENA1* is repressed by ABA, a phytohormone known to play important roles in plant immunity [47], suggest a potential role of this *trans*-NAT in plant-pathogen interaction. A further connection between *trans*-NAT, ABA and plant immunity is provided by the potential transcriptional regulatory *trans*-NAT XLOC_001125 (Fig. 2c), which is induced by ABA, and its target AT1g63350 encoding a protein belonging to the family of R proteins containing nucleotide-binding site and leucine-rich repeat (NBS-LRR) domains and participating in the plants defense to pathogens, including virus [48–50].

Beyond forming RNA:RNA double strand hybrids, lncRNAs may also form R-loops, composed of a Watson-Crick RNA-DNA hybrids and a displaced single-stranded DNA [51]. A growing number of lncRNAs have been shown to be involved in formation of R-loops either in *cis*, such as for the COOLAIR *cis*-NAT on the *FLC* locus in Arabidopsis [52] and the GATA3-AS1 lncRNA that shares a promoter region with the divergent *GAT3* gene in human [53], or in *trans* for the GAL4 lncRNA in *S. cerevisae* [54]. In the aforementioned examples, R-loop formation by lncRNAs was associated with both stimulatory and inhibitory effect of target gene expression. Formation of R-loops between lincRNAs and target gene DNA could thus be a mechanism explaining some of the associations found in the set of 101 and 81 lincRNA-HD that were either positively or negatively correlated, respectively, with changes in steady-state level of their predicted target gene.

TE are widely distributed in genomes of eukaryotes, including in Arabidopsis [55]. In humans, more then 75% of lncRNAs contain sequences originating from TE [56]. Previous study in Arabidopsis found 47 lincRNAs containing TE sequences (thus named TE-lincRNAs), with 40% of them derived from RC/Helitron TE [57]. A similar large fraction (42%) of lincRNAs identified in the present study harbored sequences to the RC/Helitron class, while sequences derived from MuDR, LTR/Copia and LTR/Gypsy were found in 18, 13 and 12% of the TE-lincRNAs. While the predominance of these classes of TE was maintained in the putative regulatory *trans*-NATs and lincRNA-DH, the overall proportion of TE-lincRNAs in these same groups increased from 25% (255 out of 1009) for all lincRNAs to 40% (52 out of 131) in lincRNA-DH and 50% (6 out of 12) in *trans*-NATs having regulatory potential on gene loci or target mRNA, respectively (Additional file 9: Table S1).

The abundance of TE in both genomic DNA and lincRNAs suggest that the formation of RNA-DNA hybrids between TE-lincRNAs and target genes containing similar TE sequences may be possible. In this context, the potential role of the TE AT1TE42205 (XLOC_000322) acting as a lincRNA-HD in the control of 13 genes (Figs. 4a-d) is interesting since all the predicted targets genes contain a sequence highly homologous to this TE in their promoter region. We have experimentally validated, using a protoplast assay, the positive and negative regulatory roles of this lincRNA-HD in *trans* on three of the 13 target genes showing high Pearson correlation coefficient, namely genes AT2G03340, AT3G23400 and AT4G04930. These data support a role for TE-lincRNAs in the regulation of target gene at the DNA level. Gene AT2G03340 encodes WRKY3, a transcription factor involved in the resistance of plants to pathogen, herbivory and salt stress [58–60]. Gene AT3G23400 encodes FIBILLIN4, a chloroplastic protein regulating plastoquinone content in plastoglobules and involved in oxidative stress [61, 62]. Although gene AT4G04930, encoding a sphingolipid desaturase, has not been directly associated with stress, plant sphingolipids have been shown to play important roles in plant responses to both biotic and abiotic stress [63–65].

Because of their capacity to inactivate genes through insertional mutagenesis, expression of TE is often regarded as harmful. Thus, TE expression is strongly suppressed by epigenetic silencing mechanisms [66]. Nevertheless, in addition to being abundantly present in lincRNAs [33, 56, 57], TE have also been found to be a prominent source of regulatory siRNAs, such as in the case of PIWI-interacting RNAs in mammals [67], as well as a potential source of miRNAs in plants [68]. Many TE in plants contain *cis*-acting elements that are responsive to stress [69] and TE-lincRNAs are often induced by various stress [33, 57, 70, 71]. Despite their abundance, only few TE-lincRNA have been identified to play a role in plants, with examples for a TE-lincRNAs contributing to stress response by an unknown mechanism [57] or to root development by acting as a miRNA sponge [72]. This work suggests that TE-lincRNAs may also contribute to the regulation of protein-coding genes containing TE in their promoter sequence and involved in stress resistance.

## Conclusions

*Trans*-NATs are one of the least characterized class of lncRNAs in eukaryotes. This work provides an analysis of lincRNAs and *trans*-NATs present in Arabidopsis that can potentially regulate protein-coding gene expression through nucleic acid base pairing. A number of differentially expressed *trans*-NATs were identified that correlated positively or negatively with the steady-state or polysome-associated levels of target gene mRNA, implicating a role of *trans*-NATs in transcriptional or translation regulation. We have also identified differentially regulated lincRNAs that can potentially regulate positively or negatively target gene expression via RNA:DNA base pairing. The implication of lincRNAs containing TE sequences in the regulation of target genes containing homologous TE sequences in their promoter was supported by transient expression in protoplast. In conclusion, this study identified lincRNAs in Arabidopsis with potential in regulating target gene expression in *trans* by both RNA:RNA and RNA:DNA base pairing and highlights lincRNAs harboring TE sequences in such activity.

## Material and methods

### Dataset

This study was based on the dataset accessible from Gene Expression Omnibus accession GSE116553. Briefly, *A. thaliana* ecotype Col-0, obtained from the Nottingham Arabidopsis Stock Center, stock number N6673 (http://arabidopsis.info/) whole seedlings grown in liquid culture for 7 days in the presence of a high (1 mM) or a low (100 μM) concentration of phosphate were analyzed along with roots and shoots from seedlings grown on agar-solidified half-strength MS medium for 10 days and then flooded for 3 h with a solution containing 5 μM IAA, 10 μM ABA, 10 μM MeJA, 10 μM ACC, or no hormone for the untreated control. For each sample, both total RNA and polysome-associated RNA was extracted and quantified by strand-specific paired-end RNAseq. Strand specific libraries were prepared using the TruSeq Stranded Total RNA kit (Illumina) and polyA^+^ RNAs were selected according to manufacturer’s instructions. The libraries were sequenced on a HiSeq 2500 Illumina sequencer. For each of the 12 experimental conditions, 3 independent biological replicates were carried out at different times. At least 30 million reads were obtained from each biological replicate.

### Identification of novel intergenic transcripts

To identify novel lincRNAs, including *trans*-NATs, the paired-end reads from the 3 replicates were pooled together and uniquely mapped to the TAIR10 genome using Hisat2 [73]. For each of the 12 conditions, the transcriptome was determined de novo with Cufflinks [74]*,* using the TAIR10.31 annotation as guide. The 12 annotation files obtained were merged using the Cuffmerge tool [74]. This transcriptome was then compared to TAIR10.31 using Cuffcompare [74], and novel transcripts not overlapping any TAIR10.31 genes (class_code_u) were considered as putative lincRNAs. This method thus removed any intronic long-coding RNAs.

### Identification of differentially expressed genes

The reads were mapped against TAIR10.31 reference genome using Hisat2 [73] and the readcount for each gene was determined using HTSeqcount [75]. Readcounts were normalized using DESeq2 [76] and genes were considered differentially expressed if fold change > 2 and adjusted *p* value < 0.1. Differences in polysome association were assessed using the Xtail package [77] and genes with a 30% increase or decrease and adjusted *p* value < 0.1 were considered differentially associated with polysomes.

### Characterization of lincRNAs

Basic features of lincRNAs including GC content or length of transcripts, average steady-state levels or polysome association were analyzed using custom functions written in Python. For the analysis of nucleotide conservation, PHASTcons scores where extracted from the 20 angiosperm genome alignment as previously described [78] and the average PHASTcons score was calculated for exonic and intronic sequences of each transcript. The presence of miRNA binding sites within lincRNAs was determined using psRNATarget server (http://plantgrn.noble.org/psRNATarget/) with an expectation <= 3 and unpaired energy (UPE) < = 25. Potential miRNA precursors were identified by comparing the cDNA sequences of lincRNAs against a database of miRNA hairpins downloaded from miRBase (http://www.mirbase.org/). The presence of potential miRNA target mimic sites was determined using custom python functions following the rules edicted in Wu et al. [79], namely, (i) perfectnucleotide pairing was required at the second to eighth positions of miRNA sequence, (ii) bulges were only permitted at the 5′ end ninth to 12th positions of miRNA sequence, and (iii) should be composed of only three nucleotides. No more than 3 mismatches or G/U pairs were allowed in pairing regions (not considering the bulge).

The presence of transposable elements within lincRNA was determined by comparing the lincRNA sequences against a database containing all transposable elements annotated in TAIR10 using Blastn with a cutoff of e value = 1e-12 and alignment length > 50.

Analysis of siRNAs that could be generated by hybridization of lincRNAs with potential targets was essentially performed according to the method described by Yuan et al. [22] using Arabidopsis small RNA dataset available on GEO. Briefly, the small reads between 18 and 28 nucleotides long were mapped to TAIR10 reference genome using bowtie. For each predicted *trans*-NAT / target pair, the length and density in small RNAs was calculated for complementary and non-complementary regions by dividing the number of mapped small reads by the length of the region using custom scripts and the python library pysam.

### Prediction of *trans*-NAT / target gene pairs

Base pair complementarity between lincRNAs and protein-coding mRNAs was determined by blasting (strand specific Blastn) each lincRNA sequence against a database made of the reverse-complement of each protein-coding mRNA. Similarly, base pair complementarity between lincRNAs and chromatin at target loci was determined by blasting lincRNA sequences (unstranded Blastn) against a database made of sequences encompassing gene body plus 2000 nucleotides upstream transcription start sites of each protein-coding gene. A gene was considered as a putative target of a lincRNA if the match between its reverse complement sequence and the sequence of the lincRNA had an e value < 1 and an alignment length > 100 nt, corresponding roughly to 70% of identity for an alignment of 100 nucleotides.

### *trans*-NATs correlated with changes in target gene mRNA polysome association (PA) or steady-state mRNA level (SS)

The *trans-*NATs potentially regulating target gene expression were identified by pairwise comparisons between whole seedlings grown under high or low Pi, roots or shoots treated with phytohormones and appropriate controls, as well as between untreated root and shoot tissues, using a series of criteria. Only the pairs *trans-*NATs */* coding gene with a normalized read count for both coding gene and lincRNA > 10 were considered. A *trans-*NATs was considered positively correlated to its predicted target gene expression if both genes were either up-regulated or down-regulated (fold change > 2 and adj. *p* value < 0.1) between the two conditions compared. It was considered negatively correlated if one partner was up-regulated while the other was down-regulated (fold change > 2 and adj. *p* value < 0.1) between the two conditions compared. To identify the potential translation regulator *trans-*NATs, we selected the pairs for which the *trans-*NAT was differentially expressed (fold change > 2 and adjusted *p* value < 0.1) and the target coding gene was differentially associated with polysomes (fold change > 1.3 and adjusted *p* value < 0.1) between the two conditions compared.

Pearson correlation coefficient between *trans-*NAT and target gene steady-state level was also calculated across the 12 experimental conditions analyzed for each candidate pair showing a positive or negative correlation. Similarly, the correlation between target mRNA PA ratio and lincRNA steady-state level was also calculated across the 12 experimental conditions for each translation regulator lincRNA candidate. The pairs with a correlation factor > 0.6 or < − 0.6 were considered as the most robust candidates.

### *trans*-NATs correlated with changes in neighbor genes steady-state mRNA level

The neighbor genes located within a windows of 10,000 nt upstream and downstream each lincRNA were identified and their pattern of expression compared to the lincRNA expression. A lincRNA and a neighbor gene were considered positively correlated if both were up or down-regulated between the two conditions compared and negatively correlated if one was up-regulated while the other was down-regulated (fold change > 2 and adj. *p* value < 0.1). As described above, Pearson correlation coefficient was also calculated for each pair lincRNA / neighbor gene.

### WGCNA clustering

Loci with a normalized read count for total RNA samples > 10 in at least 1 condition out of 12 were kept (12310 loci) and used for the weighted gene co-expression network analysis (WGCNA), performed with default parameters [80]. A total of 17 clusters of co-expression were obtained. Visual representation of the co-expression networks was done using the Cytoscape software [81].

### Data visualization

The figures showing read density from RNAseq data were generated using Integrative genomics viewer (IGV) [82] and the plot were generated using the python library matplotlib [83] and ggplot2 R package [84]. The heatmaps showing evolutionary conservation of lincRNAs were generated using the pheatmap R package.

### Transient expression by protoplast transformation

Plasmids used for protoplast transformation were assembled using BsaI-based Golden Gate cloning [85], and the final constructs contained a recombination site for Gateway™ cloning. Constructs for expression of target genes (genomic sequences including 2 kb upstream the transcription start site) included a C-terminal in-frame fusion with a foot-and-mouth disease virus (FMDV) 2A peptide, followed by fusion with a NanoLuc™ (Promega) luciferase. Additionally, an independent expression cassette driving a firefly luciferase was also included in these constructs. Constructs for expression of *trans*-NAT genes was produced without any fusion or additional expression cassette and used the Ubiquitin 4–2 promoter from *Petroselinum crispum* [86]. The sequence of the plasmids used to make the constructs are available in Genbank, accession numbers MK450602 and MK450605.

Protoplasts were produced and transformed essentially as described by Yoo et al. [87] with minor modifications. Plasmids used for transformation expressed both sense and antisense transcripts under strong and constitutive promoters, hence, to avoid artefactual gene silencing caused by high levels of dsRNA formation, we initially screened the candidates using protoplasts derived from *dcl234* mutant [88]. Selected candidates were further validated using Col0 wild-type protoplast. In brief, *dcl234* mutant or Col0 wild-type plants were grown in short photoperiod (8 h light and 16 h dark at 21 °C) for 4–5 weeks and leaves were cut with razor blades to produce 0.5–1 mm leaf strips. These were submerged in enzyme solution (1% cellulose, 0.25% macerozyme, 0.4 M mannitol, 20 mM KCl, 20 mM MES and 10 mM CaCl2), vacuum infiltrated and incubated at room temperature for 2 h. Protoplasts were harvested by centrifugation at 100 g for 3 min, washed with W5 solution (154 mM NaCl, 125 mM CaCl2, 5 mM KCl and 2 mM MES) and resuspended in MMG solution (4 mM MES, pH 5.7, 0.4 M mannitol and 15 mM MgCl2) at 1 × 10^6^ protoplast/ml. Protoplast transformation was performed by combining ~ 1.5 × 10^5^ protoplasts, 5 μg of target gene plasmid, and either 0 or 2  molar ratios of *trans*-NAT plasmid and PEG solution (40% PEG4000, 0.2 M mannitol and 100 mM CaCl2). After replacing PEG solution with W5 solution by consecutive washings, protoplasts were kept in the dark for approximately 16 h at 21 °C.

Protoplasts were harvested by centrifugation at 6000 xg for 1 min, resuspended in 1X Passive Lysis Buffer (Promega, E1941) and incubated on ice for 15 min. The lysate was cleared by centrifugation and used for luminescence quantification using a dual-luciferase system (Promega N1610), according to the manufacture’s instructions. Luminescence values for the NanoLuc™ luciferase fused to target gene was normalized against the independently expressed firefly luciferase, used as control for loading and transfection efficiency. Statistically significant differences (t-test, *p*-value < 0.05) in luciferase ratio were used to assess the effect of *trans*-NAT co-expression on the target genes.

## Additional files

Additional file 1: **Figure S1.** Analysis of the degree of overlap in lincRNAs identified in distinct studies. Venn diagram showing the number of lincRNAs identified in our study (blue area) that overlap at least partially, on the same strand, to a noncoding RNA reported in Yuan et al. (*BMC Genomics* 17, 655, 2016) (green area), in Li et al. (*Dev. Cell* 39, 508, 2016) (pink) or in Bazin et al. (*Proc. Natl. Acad. Sci. USA* 114, E10018, 2017) (red). (PDF 5992 kb)

Additional file 2: **Figure S2.** Evolutionary conservation of newly identified lincRNAs. Conservation of the nucleotide sequence of newly identified lincRNAs. The conservation index is shown on the top right and is represented has a heatmap with the colors indicating the -log of the E-value of the best blastn hit between each lincRNA and each of the 10 plant species analyzed and listed at the bottom. In the lower panel, lincRNAs were clustered into 4 groups (kmeans) and the average of the -log of the E-value is indicated for each cluster on the lower panel, using the same color code as above. (PDF 5992 kb)

Additional file 3: **Figure S3.** Analysis of co-expression networks by WGCNA clustering. A, The 17 co-expression networks constructed by WGCNA were indicated by arbitrary numbers on the left side of the table. The correlation between expression of the genes of each cluster and the different experimental conditions tested is indicated by a red to green color gradient. Bright red indicates a strong correlation, meaning that most of the genes of the cluster are up-regulated specifically in a given condition. On the opposite, dark green indicates a strong anti-correlation, meaning that most of the genes of the cluster are specifically down-regulated in a given condition. As example, genes from cluster 9 (squared in black) are induced specifically upon Pi starvation while genes associated with the cluster 14 are specifically down-regulated under the same condition. **B,** Network view of the genes belonging to the cluster 9. Only the TAIR10 genes associated with the GO term “response to Pi starvation” (blue) are shown, along with the lincRNAs identified by this study (green) or present in the TAIR10 database (red). When a lincRNA was found correlated with a putative target coding gene that was also present in the same cluster, the gene was reported and colored in purple. The nature of the correlation is indicated in legend (upper right box). The size of each octagon indicated the maximum expression level (normalized read count) across the 12 experimental conditions analysis. (PDF 5992 kb)

Additional file 4: **Figure S4.** Visual representation of sequence complementarity between segments of *trans*-NATs with their targets in mRNAs. Complementarity between segments of *trans*-NATs XLOC_001125, XLOC_003241 and AT4G16355 with their target on mRNA AT1G63350, AT4G01770 and AT2G22260, respectively, was visualized using the VARNA application (http://varna.lri.fr/). (PDF 5992 kb)

Additional file 5 **Figure S5.** Summary of the number of lincRNAs showing a correlation with a putative target or a neighboring gene. Venn diagram showing number of lincRNAs predicted to base-pair with target mRNA for which changes in lincRNA steady-state level was correlated or anti-correlated with changes in steady-state level (SS) (blue) or polysome association (PA) of target mRNA (red). LincRNAs predicted to interact with the chromatin of putative target genes level and showing a positive or negative correlation of their RNA steady-state levels are indicated by the pink area. LincRNAs coexpressed or anti-coexpressed with a neighboring gene are represented by the green area. The numbers of unique pairs lincRNA / target or neighbor genes are indicated on the Venn diagram. (PDF 5992 kb)

Additional file 6: **Figure S6.** Network view of the different types of correlations identified in this study. **A,** The nodes represent the lincRNAs whose expression was found positively or negatively correlated with at least 1 potential target gene or 1 neighboring gene. **B,** Detail of a portion of a network view highlighting the complexity of interactions between lincRNAs and their potential targets. **C**, Detail of a network view showing the multiple protein coding targets predicted for XLOC_000685 lincRNA. The 4 genes belonging to AtRLP family are encircled in blue. For A, B and C, the size of nodes indicate the maximal expression level of the gene from the 12 conditions analyzed and the color the type of gene, while the edges show the types of correlation as indicated in the left box on top in A. (PDF 5992 kb)

Additional file 7: **Figure S7.** Analysis of siRNAs in relation to trans-NATs. **A**, Histogram showing the size distribution of small reads between 18 and 28 nucleotides long mapping to regions of the putative *trans*-NATs complementary (orange) or not (blue) to their predicted target gene. **B,** Same legend as A for small reads mapping to putative target genes within regions complementary (orange) or not (blue) to *trans*-NATs. **C,** Histogram showing the distribution of enrichment scores, calculated by dividing for each *trans*-NAT (blue) small read density within regions complementary to their putative target genes by the density within non-complementary sequences. The distribution of enrichment scores for predicted *trans*-NAT targets is also reported in red. Vertical grey and black lines indicate enrichment scores of 1 and 2 on the plot. (PDF 5992 kb)

Additional file 8: **Figure S8.** Distribution of TE families in lincRNAs. Number of TE corresponding to different families present in TE-lincRNAs. (PDF 5992 kb)

Additional file 9: **Table S1.** Summary of the main characteristics of lincRNAs. (XLSX 183 kb)

Additional file 10: **Table S2.** Expression level of lincRNAs under various experimental conditions. (XLSX 123 kb)

Additional file 11: **Table S3.** LincRNAs with potential regulatory functions. (XLSX 22 kb)

## Data Availability

The data set supporting the conclusions of this article are available at the NCBI’s Gene Expression Omnibus and are accessible through GEO accession number GSE116553. The processed data tables (Additional file 9: Table S1, Additional file 10: Table S2 and Additional file 11: Table S3) are included as additional files for this article. The sequence of novel plasmids used in this study can be found at GenBank, accession numbers MK450602 and MH450605.

## Abbreviations

ABA: Abscisic acid
ACC: 1-aminocyclopropane-1-carboxylic acid
IAA: Indole-3-acetic acid
MeJA: Methyl jasmonate
NAT: Natural Antisense Transcript
PA: Polysome Association
ctrl: Untreated control
SS: Steady-State level
TE: Transposable elements
