# Supplementary figures and images for: Prediction of regulatory long intergenic non-coding RNAs acting in trans through base-pairing interactions
Source: BMC Genomics. 2019 Jul 22;20:601. doi: 10.1186/s12864-019-5946-0 (PMC6647327; doi:10.1186/s12864-019-5946-0)

Figure S1

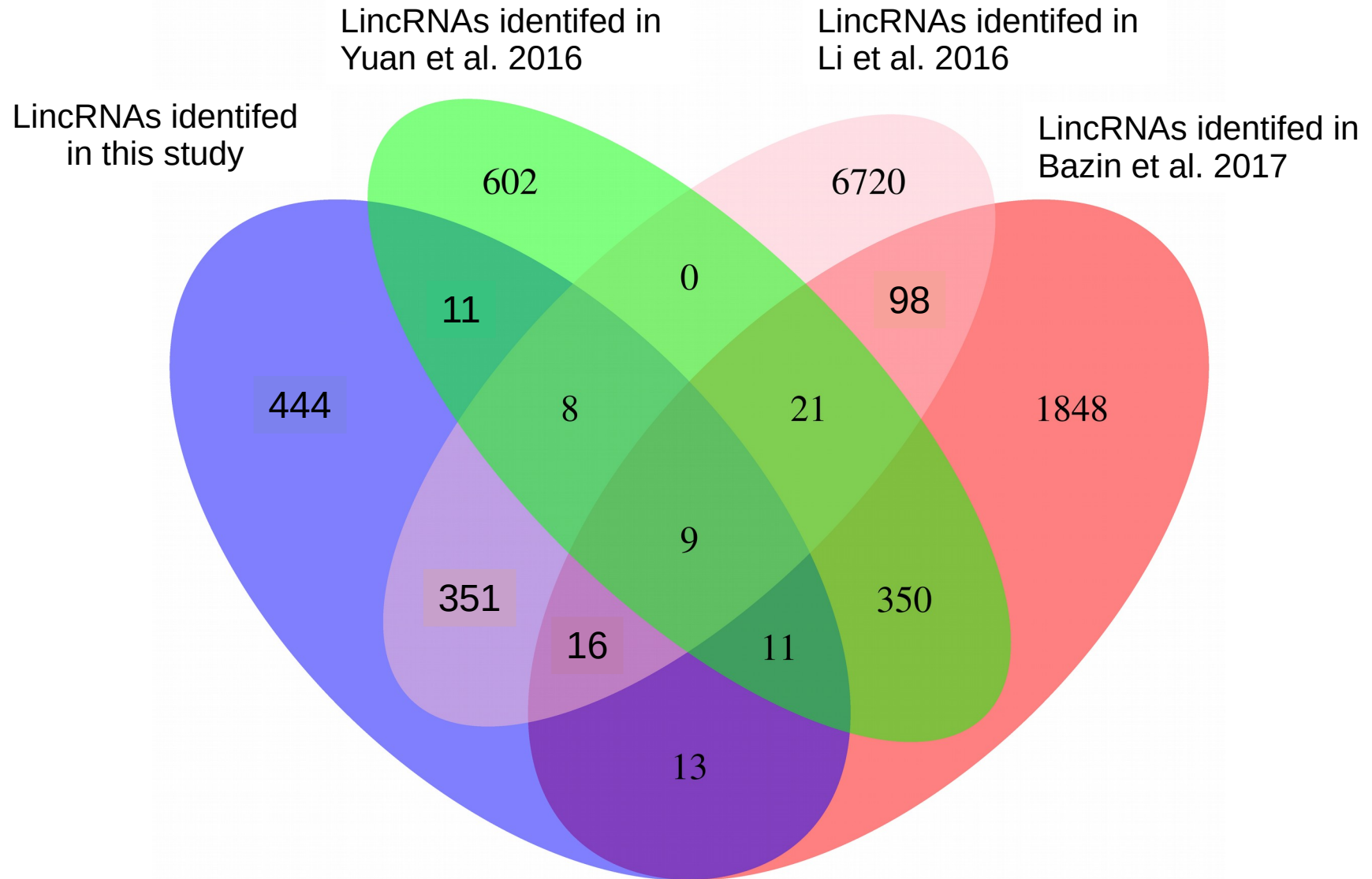

Supplement: Supplementary file 1 — Figure S1. Analysis of the degree of overlap in lincRNAs identified in distinct studies. Venn diagram showing the number of lincRNAs identified in our study (blue area) that overlap at least partially, on the same strand, to a noncoding RNA reported in Yuan et al. (BMC Genomics 17, 655, 2016) (green area), in Li et al. (Dev. Cell 39, 508, 2016) (pink) or in Bazin et al. (Proc. Natl. Acad. Sci. USA 114, E10018, 2017) (red). (PDF 5992 kb) [file 12864_2019_5946_MOESM1_ESM.pdf]

Figure S2

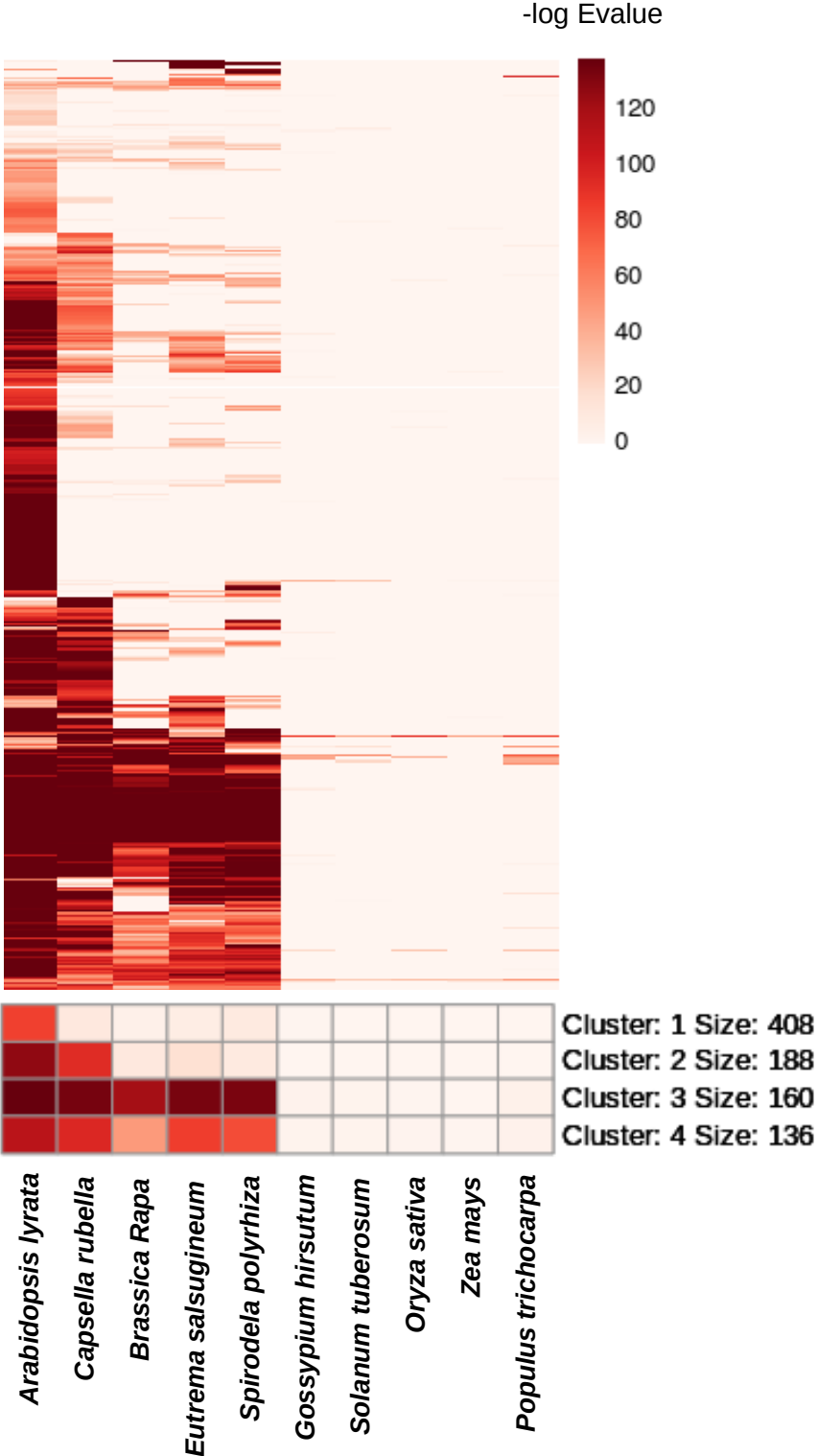

Supplement: Supplementary file 2 — Figure S2. Evolutionary conservation of newly identified lincRNAs. Conservation of the nucleotide sequence of newly identified lincRNAs. The conservation index is shown on the top right and is represented has a heatmap with the colors indicating the -log of the E-value of the best blastn hit between each lincRNA and each of the 10 plant species analyzed and listed at the bottom. In the lower panel, lincRNAs were clustered into 4 groups (kmeans) and the average of the -log of the E-value is indicated for each cluster on the lower panel, using the same color code as above. (PDF 5992 kb) [file 12864_2019_5946_MOESM2_ESM.pdf]

### Figure S3

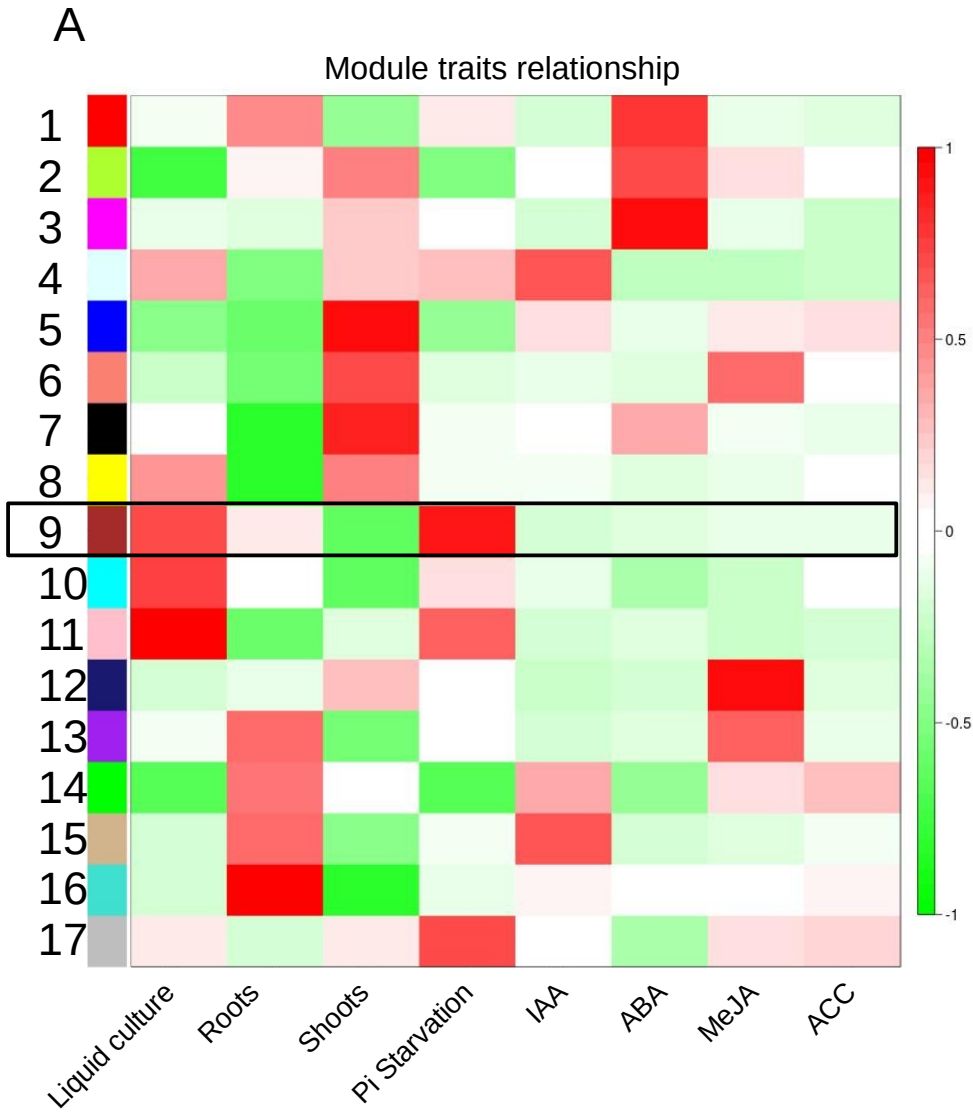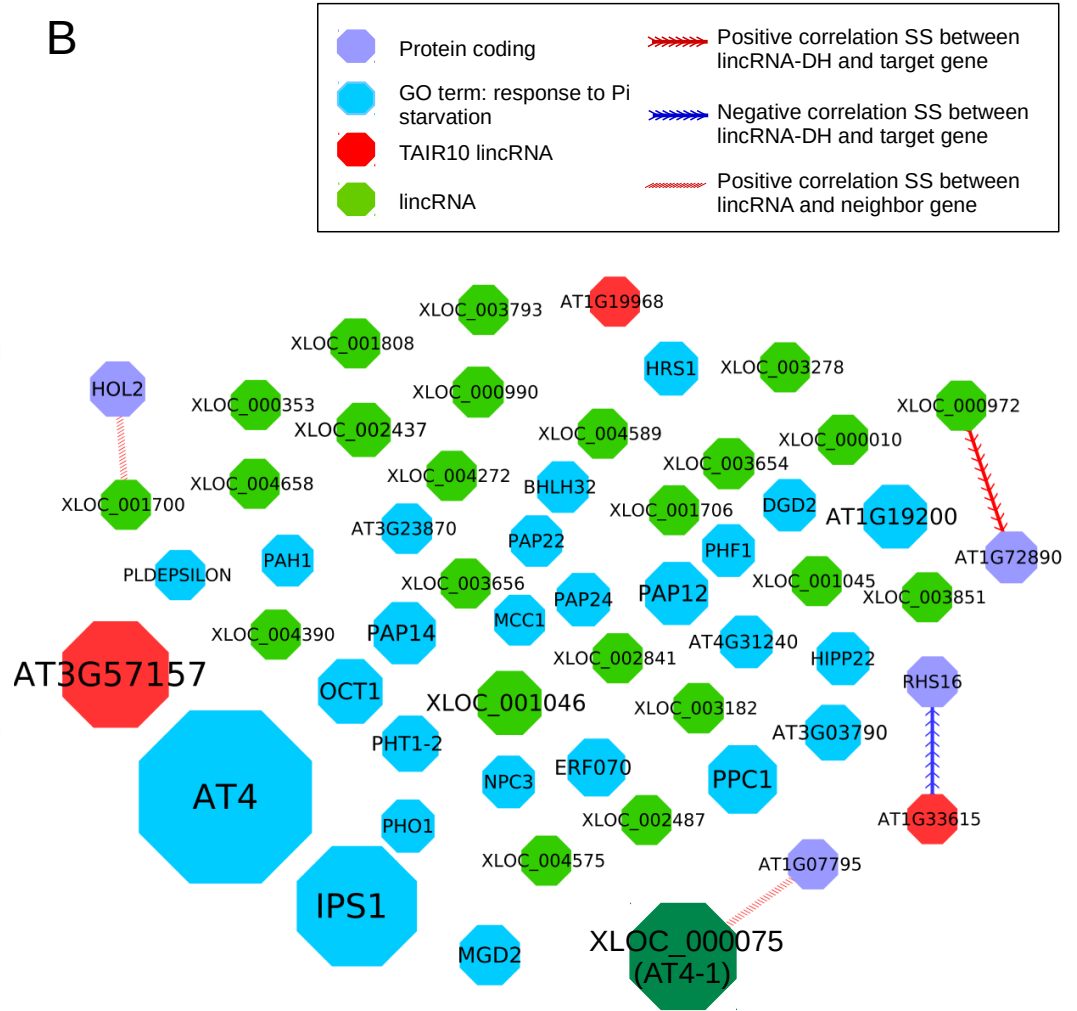

Supplement: Supplementary file 3 — Figure S3. Analysis of co-expression networks by WGCNA clustering. A, The 17 co-expression networks constructed by WGCNA were indicated by arbitrary numbers on the left side of the table. The correlation between expression of the genes of each cluster and the different experimental conditions tested is indicated by a red to green color gradient. Bright red indicates a strong correlation, meaning that most of the genes of the cluster are up-regulated specifically in a given condition. On the opposite, dark green indicates a strong anti-correlation, meaning that most of the genes of the cluster are specifically down-regulated in a given condition. As example, genes from cluster 9 (squared in black) are induced specifically upon Pi starvation while genes associated with the cluster 14 are specifically down-regulated under the same condition. B, Network view of the genes belonging to the cluster 9. Only the TAIR10 genes associated with the GO term “response to Pi starvation” (blue) are shown, along with the lincRNAs identified by this study (green) or present in the TAIR10 database (red). When a lincRNA was found correlated with a putative target coding gene that was also present in the same cluster, the gene was reported and colored in purple. The nature of the correlation is indicated in legend (upper right box). The size of each octagon indicated the maximum expression level (normalized read count) across the 12 experimental conditions analysis. (PDF 5992 kb) [file 12864_2019_5946_MOESM3_ESM.pdf]

Figure S4

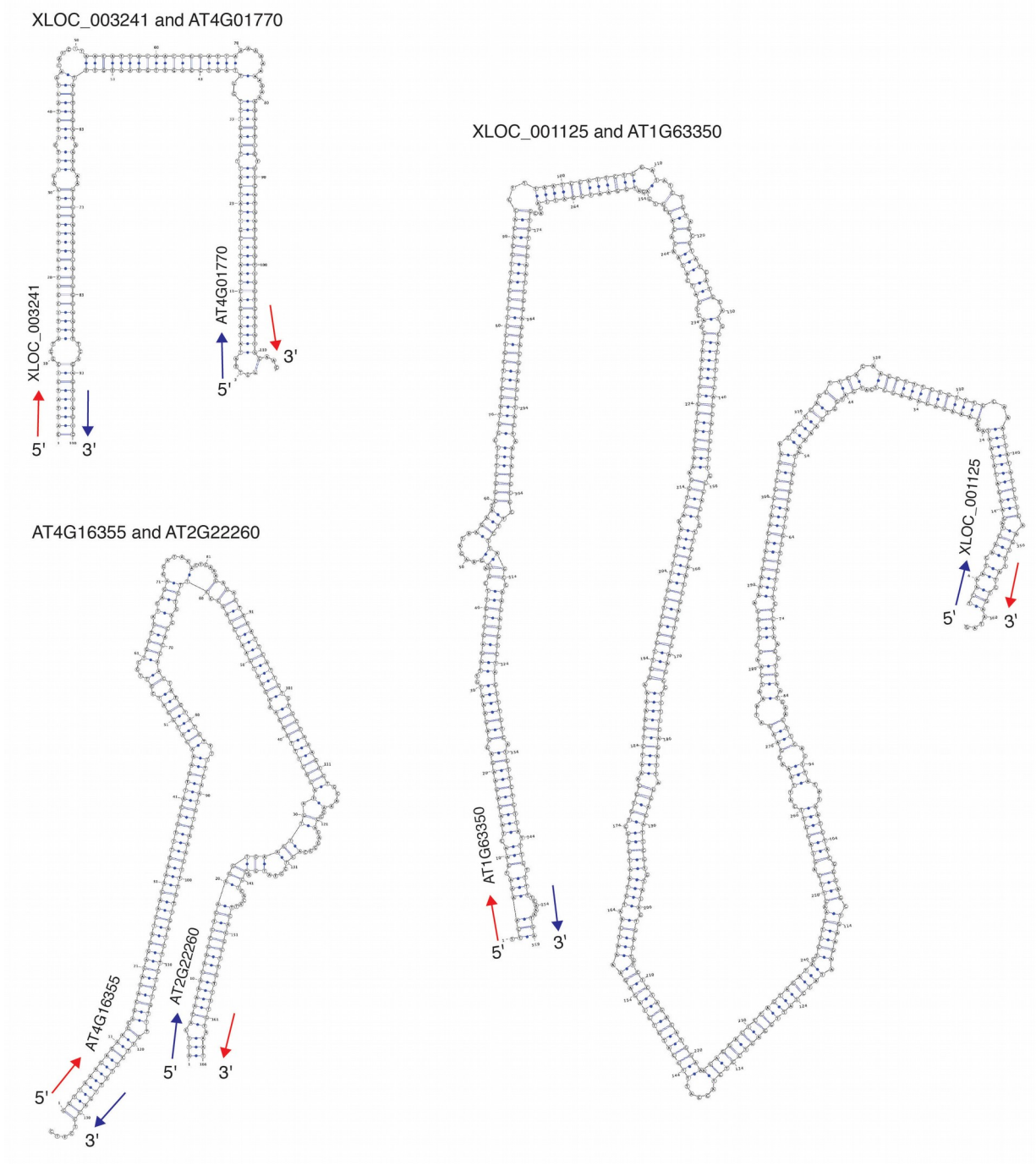

Supplement: Supplementary file 4 — Figure S4. Visual representation of sequence complementarity between segments of trans-NATs with their targets in mRNAs. Complementarity between segments of trans-NATs XLOC_001125, XLOC_003241 and AT4G16355 with their target on mRNA AT1G63350, AT4G01770 and AT2G22260, respectively, was visualized using the VARNA application (http://varna.lri.fr/). (PDF 5992 kb) [file 12864_2019_5946_MOESM4_ESM.pdf]

Figure S5

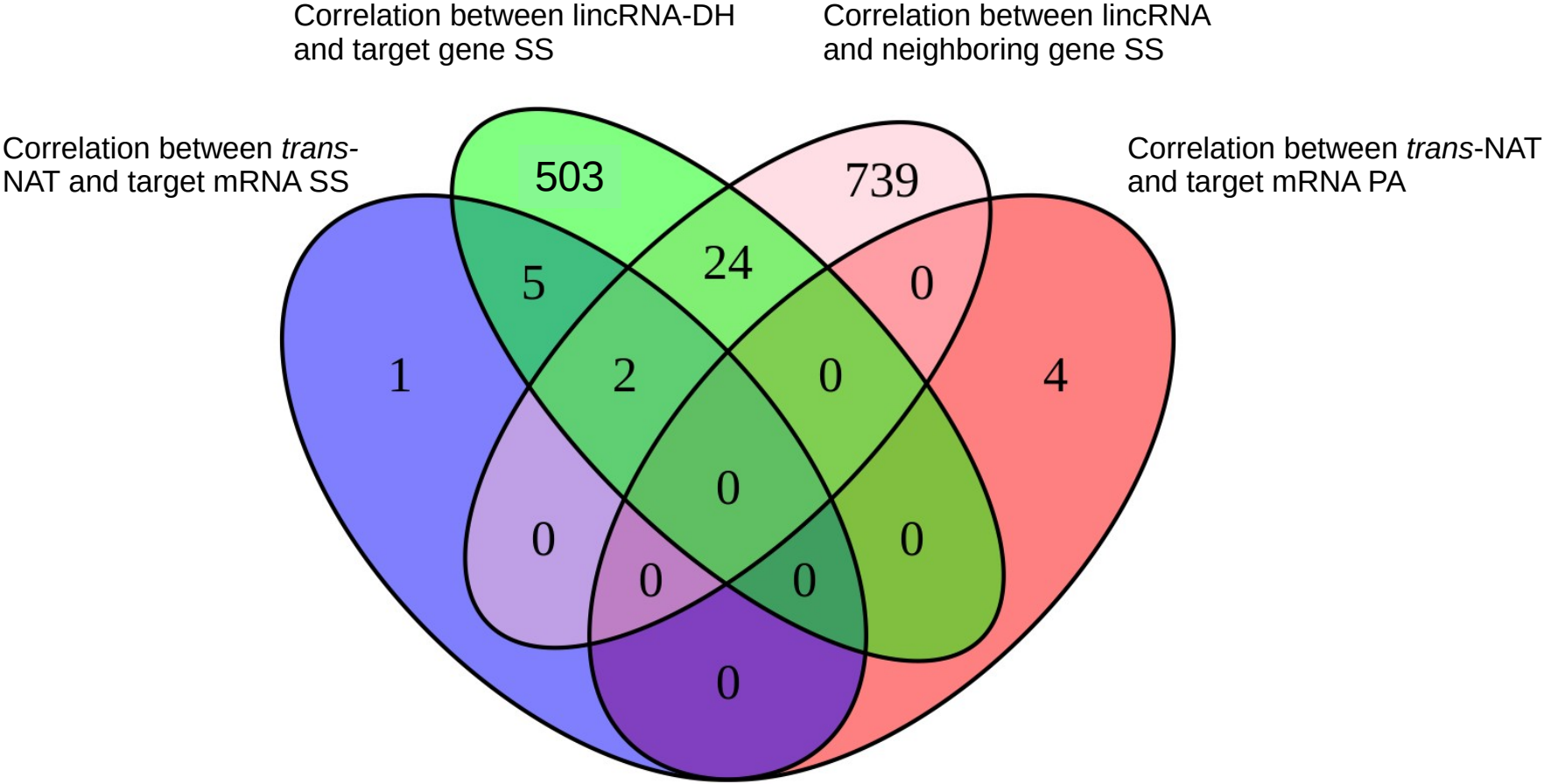

Supplement: Supplementary file 5 — Figure S5. Summary of the number of lincRNAs showing a correlation with a putative target or a neighboring gene. Venn diagram showing number of lincRNAs predicted to base-pair with target mRNA for which changes in lincRNA steady-state level was correlated or anti-correlated with changes in steady-state level (SS) (blue) or polysome association (PA) of target mRNA (red). LincRNAs predicted to interact with the chromatin of putative target genes level and showing a positive or negative correlation of their RNA steady-state levels are indicated by the pink area. LincRNAs coexpressed or anti-coexpressed with a neighboring gene are represented by the green area. The numbers of unique pairs lincRNA / target or neighbor genes are indicated on the Venn diagram. (PDF 5992 kb) [file 12864_2019_5946_MOESM5_ESM.pdf]

Figure S6

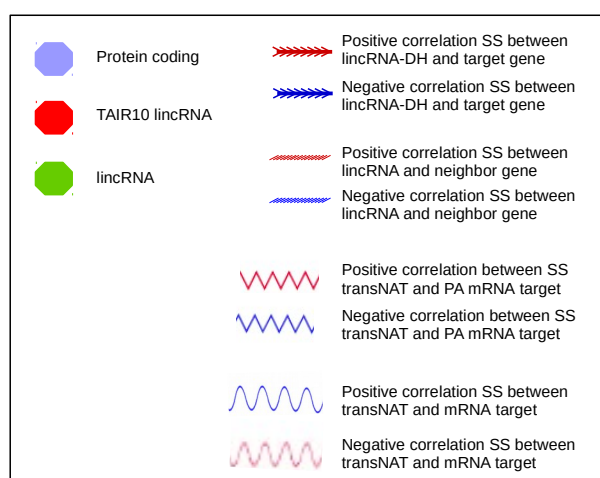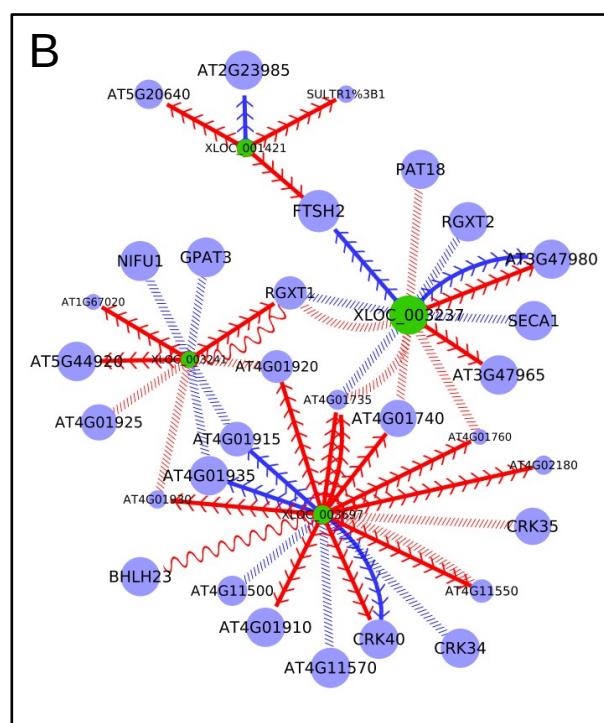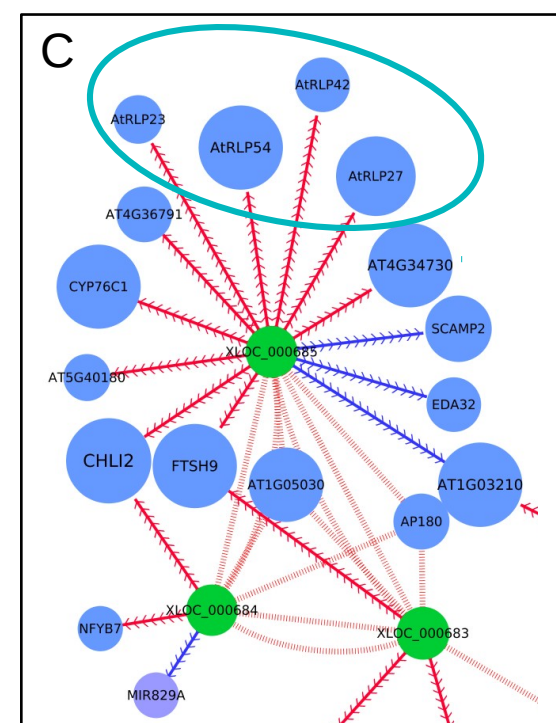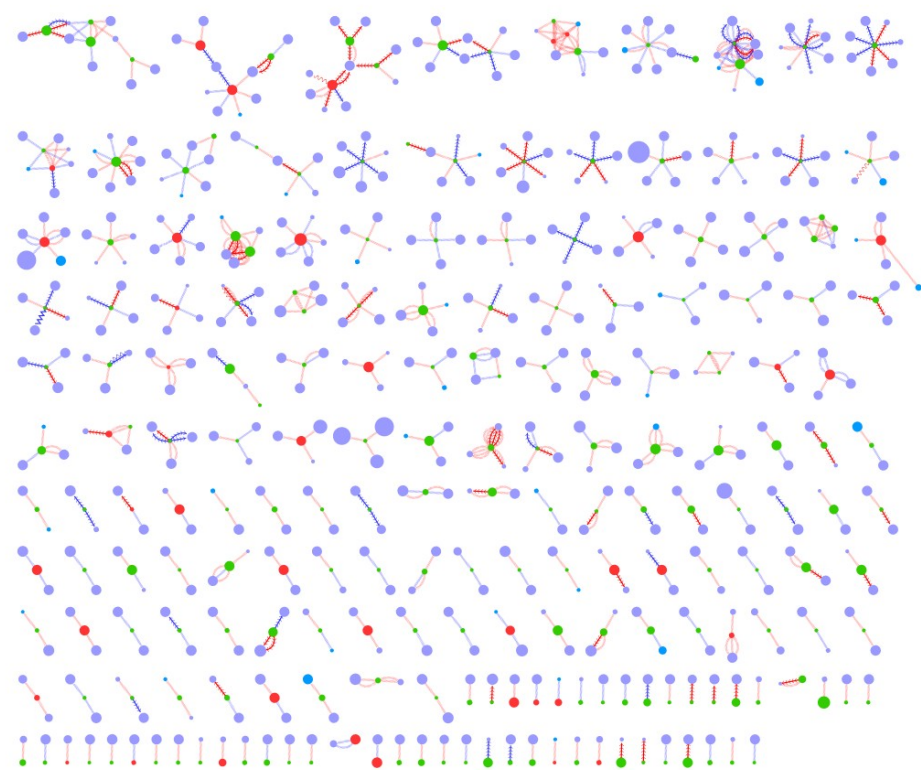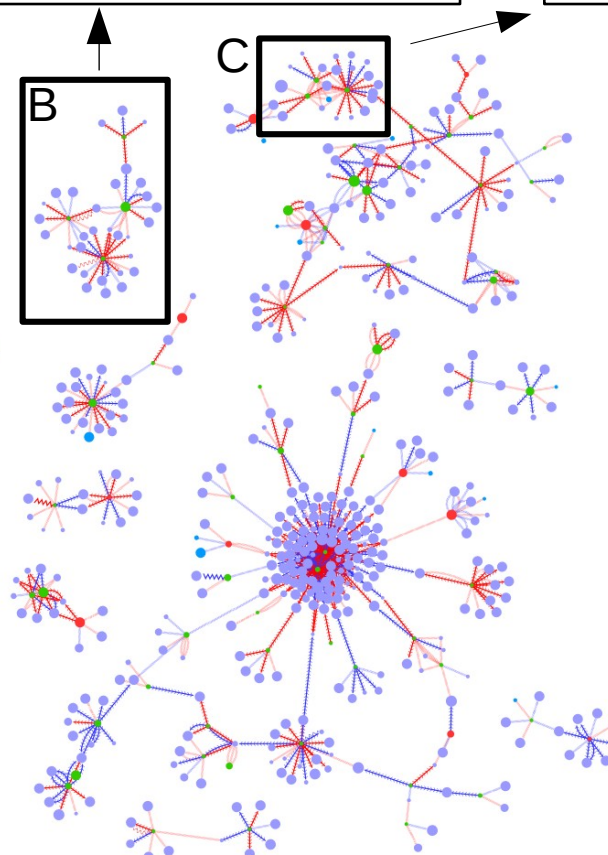

Supplement: Supplementary file 6 — Figure S6. Network view of the different types of correlations identified in this study. A, The nodes represent the lincRNAs whose expression was found positively or negatively correlated with at least 1 potential target gene or 1 neighboring gene. B, Detail of a portion of a network view highlighting the complexity of interactions between lincRNAs and their potential targets. C, Detail of a network view showing the multiple protein coding targets predicted for XLOC_000685 lincRNA. The 4 genes belonging to AtRLP family are encircled in blue. For A, B and C, the size of nodes indicate the maximal expression level of the gene from the 12 conditions analyzed and the color the type of gene, while the edges show the types of correlation as indicated in the left box on top in A. (PDF 5992 kb) [file 12864_2019_5946_MOESM6_ESM.pdf]

Figure S7

A

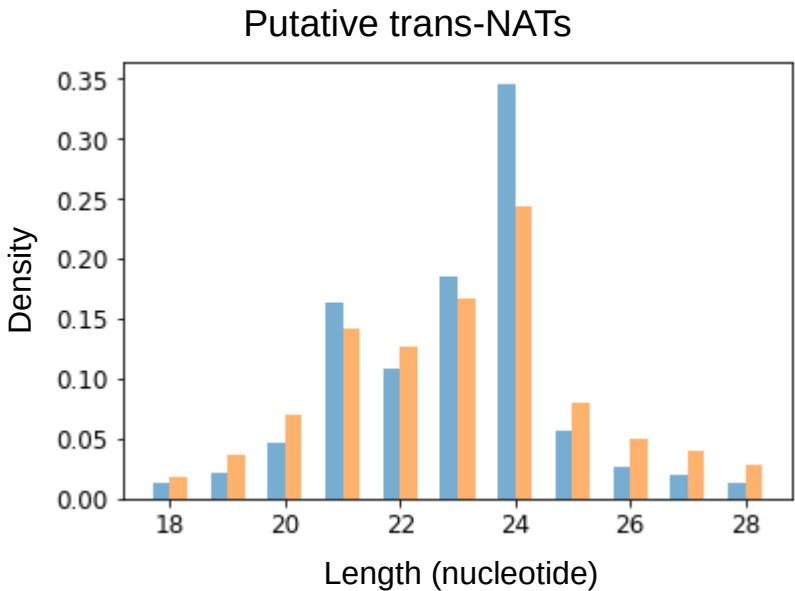

B

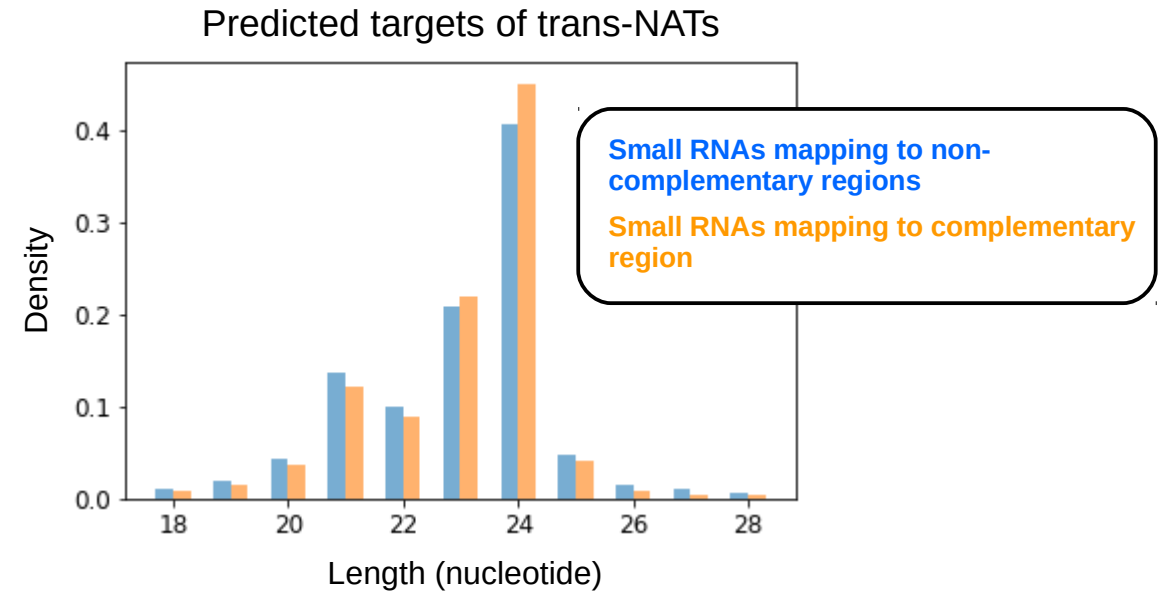

C

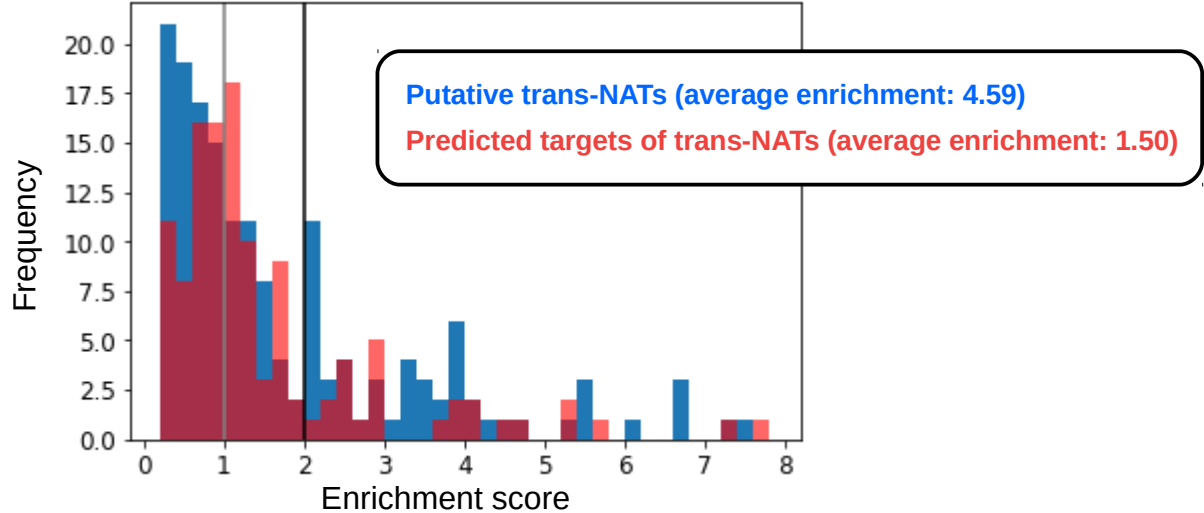

Supplement: Supplementary file 7 — Figure S7. Analysis of siRNAs in relation to trans-NATs. A, Histogram showing the size distribution of small reads between 18 and 28 nucleotides long mapping to regions of the putative trans-NATs complementary (orange) or not (blue) to their predicted target gene. B, Same legend as A for small reads mapping to putative target genes within regions complementary (orange) or not (blue) to trans-NATs. C, Histogram showing the distribution of enrichment scores, calculated by dividing for each trans-NAT (blue) small read density within regions complementary to their putative target genes by the density within non-complementary sequences. The distribution of enrichment scores for predicted trans-NAT targets is also reported in red. Vertical grey and black lines indicate enrichment scores of 1 and 2 on the plot. (PDF 5992 kb) [file 12864_2019_5946_MOESM7_ESM.pdf]

Figure S8

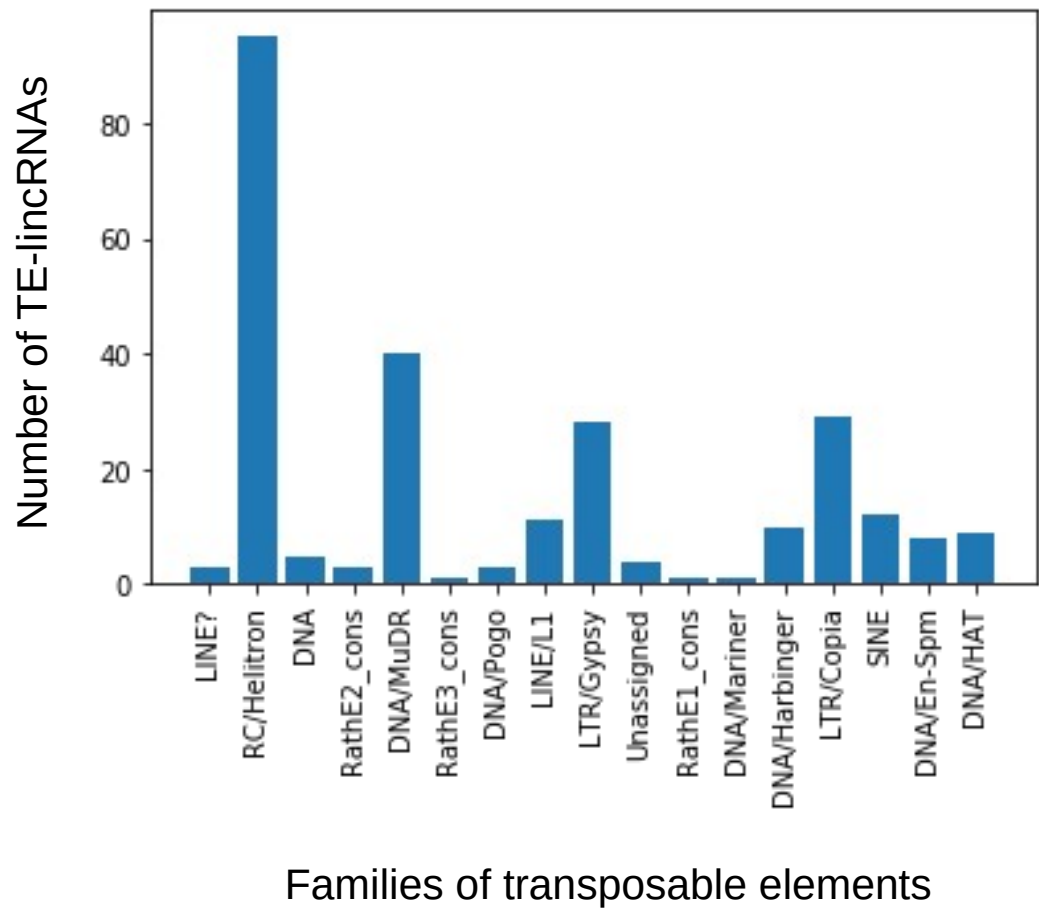

Supplement: Supplementary file 8 — Figure S8. Distribution of TE families in lincRNAs. Number of TE corresponding to different families present in TE-lincRNAs. (PDF 5992 kb) [file 12864_2019_5946_MOESM8_ESM.pdf]
